# Natural History of Germline *BRCA1* Mutated and *BRCA* Wild-type Triple-negative Breast Cancer

**DOI:** 10.1158/2767-9764.CRC-23-0277

**Published:** 2024-02-14

**Authors:** Nilesh Gardi, Rohan Chaubal, Pallavi Parab, Sunil Pachakar, Suyash Kulkarni, Tanuja Shet, Shalaka Joshi, Yogesh Kembhavi, Pratik Chandrani, Jelmar Quist, Pradnya Kowtal, Anita Grigoriadis, Rajiv Sarin, Raman Govindarajan, Sudeep Gupta

**Affiliations:** 1 Department of Medical Oncology, Tata Memorial Centre, Mumbai.; 2 Clinician Scientist Laboratory, Advanced Centre for Treatment, Research and Education in Cancer (ACTREC), Tata Memorial Centre, Navi Mumbai.; 3 Homi Bhabha National Institute, Mumbai.; 4 Department of Surgical Oncology, Tata Memorial Centre, Mumbai.; 5 Department of Radiodiagnosis, Tata Memorial Centre, Mumbai.; 6 Department of Pathology, Tata Memorial Centre, Mumbai.; 7 Cancer Bioinformatics, School of Cancer & Pharmaceutical Sciences, Faculty of Life Sciences and Medicine, King's College London, London, United Kingdom.; 8 School of Cancer & Pharmaceutical Sciences, Faculty of Life Sciences and Medicine, King's College London, London, United Kingdom.; 9 Breast Cancer Now Unit, School of Cancer and Pharmaceutical Sciences, Faculty of Life Sciences and Medicine, King's College London, London, United Kingdom.; 10 DNA sequencing Facility, Advanced Centre for Treatment, Research and Education in Cancer (ACTREC), Tata Memorial Centre, Navi Mumbai.; 11 Department of Radiation Oncology, Tata Memorial Centre, Mumbai.; 12 Samrud Foundation for Health and Research, Bangalore.

## Abstract

We report a deep next-generation sequencing analysis of 13 sequentially obtained tumor samples, eight sequentially obtained circulating tumor DNA (ctDNA) samples and three germline DNA samples over the life history of 3 patients with triple-negative breast cancer (TNBC), 2 of whom had germline pathogenic *BRCA1* mutation, to unravel tumor evolution. Tumor tissue from all timepoints and germline DNA was subjected to whole-exome sequencing (WES), custom amplicon deep sequencing (30,000X) of a WES-derived somatic mutation panel, and SNP arrays for copy-number variation (CNV), while whole transcriptome sequencing (RNA-seq) was performed only on somatic tumor.

There was enrichment of homologous recombination deficiency signature in all tumors and widespread CNV, which remained largely stable over time. Somatic tumor mutation numbers varied between patients and within each patient (range: 70–216, one outlier). There was minimal mutational overlap between patients with *TP53* being the sole commonly mutated gene, but there was substantial overlap in sequential samples in each patient. Each patient's tumor contained a founding (“stem”) clone at diagnosis, which persisted over time, from which all other clones (“subclone”) were derived (“branching evolution”), which contained mutations in well-characterized cancer-related genes like *PDGFRB*, *ARID2, TP53* (Patient_02), *TP53*, *BRAF, BRIP1*, *CSF3R* (Patient_04), and *TP53, APC*, *EZH2* (Patient_07). Including stem and subclones, tumors from all patients were polyclonal at diagnosis and during disease progression. ctDNA recapitulated most tissue-derived stem clonal and subclonal mutations while detecting some additional subclonal mutations. RNA-seq revealed a stable basal-like pattern, with most highly expressed variants belonging to stem clone.

**Significance:**

In germline *BRCA1* mutated and *BRCA* wild-type patients, TNBC shows a branching evolutionary pattern of mutations with a single founding clone, are polyclonal throughout their disease course, and have widespread copy-number aberrations. This evolutionary pattern may be associated with treatment resistance or sensitivity and could be therapeutically exploited.

## Introduction

Triple-negative breast cancer (TNBC) is an aggressive disease characterized by the lack of expression of estrogen receptor (ER), progesterone receptor (PR), and *HER2*/*neu* receptor (HER2). Endocrine and HER2 targeted therapies are ineffective in these patients. Thus, surgery, radiotherapy, and chemotherapy remain the standard treatment for this disease. Many patients with TNBC experience locoregional and/or distant relapse after primary treatment, with short post-relapse survival and acquisition of resistance to multiple drugs. The characteristic clinical features of TNBC include younger age, higher probability of germline genetic predisposition, propensity for early relapse, involvement of visceral organs, and fulminant course in patients with metastatic disease (1, 2).

Gene expression profiling studies have shown that 75%–80% of TNBC belong to the basal-like intrinsic subtype with a pattern similar to normal basal/myoepithelial cells, including expression of basal cytokeratins and *EGFR* (3). Recent large-scale studies have genomically profiled breast cancers, including TNBC (4–7). These and other studies have classified TNBC into subtypes with distinct genomic, transcriptomic and copy-number profiles, and clinical outcomes (8–13). These studies have shown that basal-like TNBC are characterized by high levels of genomic instability leading to widespread, genome-wide copy-number aberrations, and marked interpatient heterogeneity in mutational profiles. These studies using predominantly treatment-naïve TNBC samples have provided important insights about its landscape and molecular drivers, but are inadequate for explaining the subsequent disease course.

Notably, the drivers of cross-sectional (across patients) and sequential (“in a patient over time”) heterogeneity may be different, with the latter arising under the pressure of drugs used sequentially (14). Some landmark studies have attempted to resolve the clonality of TNBC at a snapshot in time or multiregional snapshots in time (14–17), while others have characterized its evolution under therapeutic pressure (18), or in patient-derived xenograft models (19–22). Some studies have evaluated the evolutionary pattern of TNBC by comparing primary and metastatic tumors obtained from different organs at autopsy (23, 24) and identified considerable similarity in genomic aberrations between them within individual patients.

The sequential evolution of tumor through its life history from initial diagnosis in a non-metastatic stage to the development of metastatic disease and subsequently repeated disease progressions in a single patient is less well reported. This could provide vital insights into adaptation mechanisms and escape from different treatments. It could also help elucidate whether the founding clone(s) (“trunk”) persists through the life history of a tumor, with evolution mainly comprising “branches” from the “trunk,” or there is clonal extinction and the emergence of new clones.

We performed a prospective sequential sampling of somatic tissue and circulating tumor DNA (ctDNA) from 3 patients with TNBC over their disease course to evaluate clonal evolution under the selection pressure of chemotherapy drugs.

## Materials and Methods

### Study Design, Patients, and Samples

The study was approved by the Institutional Ethics Committee of Tata Memorial Centre, Mumbai, India as IEC study protocol 151 and registered in the Clinical Trials Registry—India (CTRI/2016/11/007430). This study was conducted in accordance with the Declaration of Helsinki. Patients were included in the study after obtaining written informed consent, including consent for publication. This was an ambispective (prospective and retrospective) study with respect to sample and data collection. Patients were eligible if they had histopathologically proven breast cancer, which was negative for ER, PR, and HER2 by IHC or FISH, if required. The included patients were those who had their first relapse (local or distant) after prior curative treatment with surgery, (neo) adjuvant chemotherapy, with or without radiotherapy. Tumor specimens [fresh-frozen or formalin-fixed and paraffin-embedded (FFPE) tissue] from initial diagnosis and surgery were required to be available in the hospital tumor tissue repository.

After recruitment in the study, patients underwent blood sampling and tumor tissue sampling from the most accessible site of relapse. Briefly, multiple cores of fresh tumor biopsy were stored in three to four tubes of an RNA preservative (RNAlater) at 2°C–8°C for 12–16 hours, followed by −80°C until further analysis. The stored samples were subjected to whole-exome sequencing (WES), whole transcriptome sequencing (RNA-seq), high-depth targeted resequencing, and SNP array for copy-number variation (CNV), as described below and in the Supplementary Materials and Methods. Routine histopathologic evaluation, including IHC analysis for ER, PR, HER2, and tumor content, was performed on a few cores of the fresh biopsy.

Blood (12–15 mL in ethylenediaminetetraacetic acid (EDTA) tube, except in 1 patient at one timepoint in whom only 3.6 mL was available) was separated into buffy coat and plasma using cold centrifugation at 820 × *g* for 10 minutes at 4°C followed by 20,000 × *g* for 10 minutes at 4°C, which were then stored at −80°C until further analysis. Germline DNA from buffy coat was subjected to WES, high-depth targeted resequencing, SNP array for CNV, and custom amplicon next-generation sequencing (NGS) assay for hereditary predisposition genes, as described below and in Supplementary Materials and Methods. Plasma was used for ctDNA analysis as described below and in the Supplementary Materials and Methods.

The patients were treated with chemotherapy per standard practice and followed up with clinical and radiological evaluation. At the time of documented progression, fresh tumor biopsy and blood samples were again obtained and stored as described above. These steps were repeated at the time of each documented disease progression after subsequent lines of treatment until the last disease progression before death.

FFPE tissues obtained at the time of initial diagnosis and surgery (after neoadjuvant chemotherapy) were used to extract DNA and subjected to WES and high-depth targeted sequencing as described below and in the Supplementary Materials and Methods.

### DNA-based Assays

DNA from fresh-frozen and buffy coat samples was extracted using the Qiagen DNA mini kit (catalog no./ID: 51306) and from FFPE samples using Qiagen DNA FFPE kit (catalog no./ID: 56404), as per manufacturer's protocols. Agilent V4+UTR (71M) exome capture was used to perform WES at an average depth of 200X (tumor samples) and 100X (buffy coat DNA). Somatic mutations from WES analysis of each patient's tumor tissue (all samples) were pooled to design a custom targeted amplicon panel assay, which was performed on DNA from all tumor samples, corresponding WBC, and ctDNA from plasma. The OncoScan assay (Affymetrix, Thermo Fisher Scientific, catalog no./ID: 902293), a high-density microarray platform, was used for SNP profiling on DNA extracted from all fresh-frozen and buffy coat samples, as per the manufacturer's protocol. Insufficient DNA was available from FFPE samples for this assay and WES data were used to infer copy numbers in these samples using VarScan2 (RRID: SCR_006849) and Sequenza (RRID:SCR_016662) tools (details in Supplementary Materials and Methods). A total of 6 mL of plasma from each blood sample was used to extract ctDNA using the Circulating Nucleic Acid kit (catalog no./ID: 55114) from Qiagen, according to the manufacturer's instructions. Extracted ctDNA from each plasma sample was eluted into 55 µL of AVE buffer and stored at −20°C. The concentration of extracted ctDNA was determined using Qubit dsDNA HS (High Sensitivity) Assay Kit (Invitrogen), and its size distribution was assessed using a fragment analyzer. In addition, a custom amplicon targeted NGS assay for germline variants in genes with an established role in hereditary cancers was performed on buffy coat DNA, as reported earlier (25). WES capture and library preparation, targeted custom amplicon deep sequencing assay for pooled somatic variants, panel germline variant NGS assay design, and targeted sequencing and Sanger validation of germline variants are described in Supplementary Materials and Methods.

### RNA-based Assays

RNA-seq could only be performed on prospectively collected tumor samples because insufficient tumor was available for this assay in FFPE blocks. RNA was extracted from fresh-frozen samples using the Qiagen RNAeasy mini kit (catalog no./ID: 74004) per the manufacturer's instructions. RNA concentration was measured using Qubit Fluorometer, and the 28S/18S ratio was determined using Agilent 2100 bioanalyzer. RNA integrity number was checked using the Agilent Bioanalyzer and was >7 for all fresh-frozen samples. NGS Libraries for RNA-seq were prepared using the manufacturer's instructions (Illumina True-Seq mRNA Sample Preparation Kit) and are described in detail in Supplementary Materials and Methods.

### NGS and its Analysis

High-throughput sequencing was independently performed for each captured library to ensure that each sample met the desired coverage. Sequences were generated as 150 bp paired-end reads for WES (200X depth), 100 bp paired-end reads for targeted custom amplicon deep sequencing assay for pooled somatic variants (30,000X), and 150 bp paired-end reads for whole transcriptome libraries (∼60 million reads per sample). Raw image files were processed for base calling using the Illumina base-calling Software 1.7 (RRID:SCR_014332) with default parameters. Bioinformatics analysis for CNV analysis using SNP profiling, copy-number inference from WES, somatic variant calling from WES and RNA-seq data, germline variant calling from white blood cell (WBC) DNA, and deep sequencing validation at 30,000X from targeted custom amplicon assay, are described in detail in Supplementary Materials and Methods. We categorized the variants chosen to be validated by ultra-high depth sequencing data (*n* = 1006) into four tiers as described in Supplementary Materials and Methods.

### Clonality and Phylogenetic Analysis

We used the PyClone (RRID:SCR_016873; ref. 26) software for clonality analysis. A clone was defined as a cluster of mutations with the same allelic frequency at each timepoint and whose allelic frequencies tracked together over time. Any clone that persisted throughout the disease course was termed the *“stem clone”* or *“founder clone*.*”* Additional mutations gained by the founder clone resulted in “subclones.” Clonal maps of each sample were constructed, and clonal evolution was inferred from sequential samples in each patient. Briefly, allele frequency obtained from deep sequencing of sequential samples in each patient was integrated with allele-specific CNV information and tumor content (inferred from sequencing data), and inputted into the PyClone software. We ran the PyClone algorithm for 10,000 iterations using the beta-binomial model, with other options kept at default settings. PyClone estimates each mutation's clonal prevalence (CP) and clusters mutations into various groups (“clones”). Clusters with more than one mutation were considered further. CALDER (27) was used to infer an evolutionary phylogenetic tree for each patient, and TimeScape (28) was used for visualization.

### Data Availability Statement

All raw data are deposited in ArrayExpress with following IDs: E-MTAB-11379 (Exome sequencing), E-MTAB-11375 (Transciptome sequencing), E-MTAB-11375 (SNP array data), and E-MTAB-11376 (ultra-deep sequencing). Other data generated in this study are available within the article and its Supplementary Data files.

## Results

### Clinical Characteristics and Sample Accrual

Three patients with TNBC, ages 29 years, 34 years, and 28 years, were included in this study between December 23, 2015, and March 17, 2016. The important clinical characteristics of the patients are depicted in Table 1. All 3 patients received anthracycline-based neoadjuvant chemotherapy followed by surgery, and all patients also received paclitaxel, 1 in neoadjuvant setting and 2 after surgery.

**TABLE 1:** Detailed description of patient samples and applied genomic assays

Patient	Retrospective/Prospective	Month/Year	Timepoint	Material type	Tumor percent reported by pathologist	Event	Sample notation	Whole-exome sequencing	SNP array	Transcriptome	Custom amplicon deep sequencing	Germline panel sequencing	Sanger sequencing
Patient_02	Retrospective	May-15	Diagnosis	FFPE tissue	65%	Diagnostic biopsy	02_Bio	Yes	—	—	—	—	—
	*Neoadjuvant chemotherapy with four cycles of cyclophosphamide plus doxorubicin plus 5-fluorouracil (May 16, 2015 to July 23, 2015) followed by four cycles of paclitaxel plus carboplatin (August 13, 2015 to October 15, 2015)*												
	Retrospective	Nov-15	Surgery	FFPE tissue	85%	Surgical specimen	02_Sur	Yes	—	—	Yes		
	*None*												
	Prospective	Dec-15	First relapse	Blood		Germline control	02_Blood	Yes	Yes	—	Yes	Yes	Yes
				Fresh frozen tumor tissue	80%	First progression	02_Rec1	Yes	Yes	Yes	Yes	—	—
				ctDNA		ctDNA at first progression	P2R1	—	—	—	Yes	—	—
	*Three cycles of oral capecitabine (December 28, 2015 to February 21, 2016)*												
	Prospective	Mar-16	Second relapse	Fresh-frozen tumor tissue	80%	Second progression	02_Rec2	Yes	Yes	Yes	Yes	—	—
				ctDNA		ctDNA at second progression	P2R2	—	—	—	Yes	—	—
	*Four cycles of gemcitabine plus cisplatin (March 02, 2016 to June 6, 2016)*												
	Prospective	Jun-16	Third relapse	Fresh-frozen tumor tissue	30%	Third progression	02_Rec3	Yes	Yes	Yes	Yes	—	—
				ctDNA		ctDNA at third progression	P2R3	—	—	—	Yes	—	—
	*Whole brain radiotherapy (July 12, 2016 to July 16, 2016)* and *oral cyclophosphamide plus oral methotrexate (not started)*												
	—	Aug-16	Death	—		Death	—	—	—	—	—	—	—
Patient_04	Retrospective	Aug-14	Diagnosis	FFPE tissue	75%	Diagnostic biopsy	04_Bio	Yes	—	—	—	—	—
	*Neoadjuvant chemotherapy with four cycles of cyclophosphamide plus doxorubicin plus 5-fluorouracil (September 17, 2014 to November 18, 2014)*												
	Retrospective	Jan-15	Surgery	FFPE tissue	70%	Surgical specimen	04_Sur	Yes	—	—	—	—	—
	*Four cycles of paclitaxel (February 07, 2015 to April 11, 2015) followed by locoregional radiotherapy (May 25, 2015 to June 12, 2015)*												
	Prospective	Dec-15	First relapse	Blood		Germline control	04_Blood	Yes	Yes	—	Yes	Yes	Yes
				Fresh-frozen tumor tissue	80%	First progression	04_Rec1	Yes	Yes	Yes	Yes	—	—
				ctDNA		ctDNA at first progression	P4R1	—	—	—	Yes	—	—
	*Three cycles of gemcitabine plus carboplatin (January 27, 2016 to March 10, 2016)*												
	Prospective	Apr-16	Second relapse	Fresh-frozen tumor tissue	80%	Second progression	04_Rec2	Yes	Yes	Yes	Yes	—	—
				ctDNA		ctDNA at second progression	P4R2	—	—	—	Yes	—	—
	*Injection oxaliplatin plus oral cyclophosphamide plus oral capecitabine (April 01, 2016 to May 03, 2016)*												
	—	Jun-16	Death	—		Death	—	—	—	—	—	—	—
Patient_07	Retrospective	Jul-14	Diagnosis	FFPE tissue	65%	Diagnostic biopsy	07_Bio	Yes	—	—	Yes	—	—
	*Neoadjuvant chemotherapy with four cycles of cyclophosphamide plus epirubicin plus 5-fluorouracil (August 04, 2014 to October 07, 2014)*												
	Retrospective	Jan-15	Surgery	—		Surgical specimen	—	—	—	—	—	—	—
	*12 cycles of weekly paclitaxel (December 2014 to February 2015) followed by locoregional radiotherapy (February 2015 to March 2015)*												
	Prospective	Mar-16	First relapse	Blood		Germline control	07_Blood	Yes	Yes	—	Yes	Yes	Yes
				Fresh-frozen tumor tissue	60%	First progression	07_Rec1	Yes	Yes	Yes	Yes	—	—
				ctDNA		ctDNA at first progression	P7R1	—	—	—	Yes	—	—
	*Six cycles of gemcitabine plus carboplatin plus oral bicalutamide (March 12, 2016 to July 08, 2016)*												
	Prospective	Aug-16	Second relapse	Fresh-frozen tumor tissue	70%	Second progression	07_Rec2	Yes	Yes	Yes	Yes	—	—
				ctDNA		ctDNA at second progression	P7R2	—	—	—	Yes	—	—
	*Three cycles of oral capecitabine (August 08, 2016 to September 26, 2016)*												
	Prospective	Oct-16	Third relapse	Fresh-frozen tumor tissue	70%	Third progression	07_Rec3	Yes	Yes	Yes	Yes	—	—
				ctDNA		ctDNA at third progression	P7R3	—	—	—	Yes	—	—
	*Three cycles of cyclophosphamide plus methotrexate plus FU (October 03, 2016 to November 07, 2016)*												
	—	Nov-16	Death	—		Death	—	—	—	—	—	—	—

All patients had residual tumors after neoadjuvant chemotherapy and relapsed at 1 month, 11 months, and 16 months, respectively, from the date of surgery. All patients received carboplatin during their disease course, 1 during neoadjuvant treatment and 2 for metastatic disease, and all had two episodes of disease progression after the first relapse. The 3 patients died at 9 months, 6 months, and 7 months after experiencing the first relapse. The disease events, collection timepoints, nature of the sample, intermediate chemotherapy drugs, and the genomic assays applied on each sample in the 3 patients are listed in Table 1. The sites of prospective tumors biopsy, their histopathologic details including nodal involvement and tumor grade are detailed in Supplementary Table S1. In total, we were able to accrue 13 sequential tissue samples (five FFPE, eight fresh-frozen), eight sequential ctDNA samples at each relapse/progression, and three germline WBC samples at first relapse, from 3 patients. DNA from eight fresh-frozen tissues and three germline samples were subjected to WES, SNP array, and deep sequencing assays. The eight ctDNA samples were subjected to deep sequencing assays. RNA from eight fresh-frozen tissues was also subjected to RNA-seq assays. The three germline DNA samples were additionally subjected to a 35-gene NGS germline assay and Sanger sequencing for *BRCA* mutations.

### Tumor Subtyping

IHC on tumor samples obtained at each timepoint confirmed all samples in the 3 patients to be ER, PR, and HER2 negative. Analysis of genome-wide RNA-seq data by AIMS (Absolute Assignment of breast cancer intrinsic Molecular Subtype; ref. 16) on fresh tumor samples from sites of relapse or progression confirmed that all of them belonged to the basal-like category.

### Germline Mutations in Patients

WES on the germline DNA from Patient_02 (*BRCA1* c.5035delC, heterozygous deletion) and Patient_07 *(BRCA1* c.4676-1 G>C, heterozygous splice site) suggested the presence of pathogenic mutations in *BRCA1* gene. The third patient had wild-type *BRCA1* and *BRCA2* status in her germline. Details of mutations and significance are provided in Supplementary Data. These findings were confirmed using targeted 35-gene germline panel testing with NGS and with Sanger sequencing (Supplementary Fig. S1).

### Somatic Mutations and Mutational Signatures from WES

**FIGURE 1:**
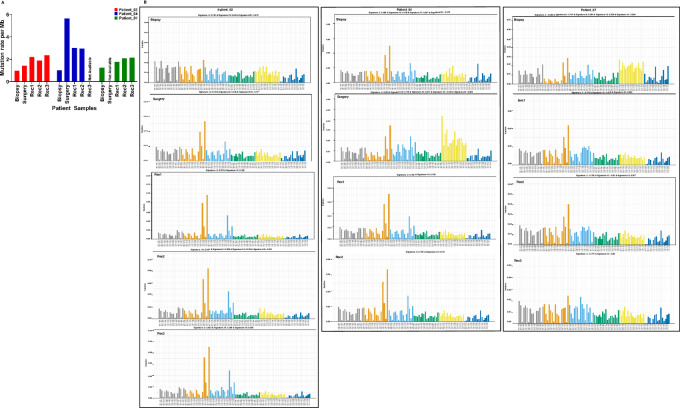
Mutation rate and mutational signatures over disease course. **A,** X-axis represents samples, Y-axis represents mutations per MB. The mutation rate in pre-therapy samples is lower than that in post-therapy samples. **B,** X-axis represents 96 possible classes of mutations. Y-axis represents the fraction of these classes. The sample name is shown in each box.

The median coverage in WES was 264.20X for fresh-frozen samples, 150.26X for buffy coat samples, and 107.62X for FFPE samples. The median number of mutations per megabase (Mb) in somatic tumor samples (*n* = 13) was 1.59 (range: 0.97–5.65), which is concordant with the known mutation rate in TNBC (Fig. 1A).

We identified a total of 1,198 somatic alterations (1,083 unique somatic point mutations and 115 indels) in 1,101 genes (Supplementary Table S2) across 13 samples (eight fresh-frozen, and five FFPE) from 3 patients (349 in Patient_02, 557 in Patient_04, and 192 in Patient_07, respectively), which were further ultra-high depth sequenced at a median coverage of 30,000X for validation. Consistent with previous reports, no mutation was common in all 3 patients (14), except one gene, *TP53.* Mutational signature 3 (associated with *BRCA1* mutation) and mutational signature 13 were enriched in all tumor samples from all patients (Fig. 1B). In addition, some signatures were found at low fractions in only one of the samples in each patient. These were signatures 1A, 9, 16, 18, and 19 in one sample each in Patient_02, signatures 12, 16, 17, R3, and 21 in one sample each in Patient_04, and signatures 8, 9, 12, 15, and 19 in one sample each in Patient_07.

### Variant Validation and Identification of Low Allelic Prevalence Variants Using Targeted Deep Sequencing

Deep sequencing allowed us to identify low allele frequency subclonal mutations across various samples, which could have been missed in WES. Deep sequencing could not be performed on three FFPE samples (02_Bio, 04_Bio, 04_Sur) due to inadequate tissue. Of the 1,083 mutations shortlisted from WES, 77 could not be sequenced at high depth, while we could successfully design primers for 1,006 variants (Supplementary Table S3). There was concordance in 834 of the 1,006 mutations (82.9%) between exome sequencing and deep sequencing. There was a very high concordance in allele frequency of mutations between WES and targeted deep sequencing for fresh-frozen samples (99%) and moderately high concordance for FFPE samples (78%). Variant allele frequencies from exome and deep sequencing data were strongly correlated with *R* > 0.9 for all samples (Supplementary Fig. S2), which suggests that WES can effectively capture variant allele frequencies for almost all single-point mutations. There was discordance in 172 of 1,006 unique single point mutations in that some of them were not detected by WES but were detected by deep sequencing in that sample (Supplementary Fig. S3). This suggests that these mutations may not have been adequately covered in WES or that these were subclonal mutations detectable only by high-depth sequencing. There were 25 Tier 1 variants, 41 Tier 2 variants, 286 Tier 3 variants, and 619 Tier 4 variants across 13 samples from the 3 patients.

### Copy-number Analysis

Because DNA from baseline FFPE samples was exhausted, we could not subject them to SNP array analysis and only the eight fresh-frozen samples collected prospectively at the time of disease progression were subjected to this analysis. Reduced Segment (RS) analysis identified changes in 98.3% of the genome across eight samples (mean values: focal amplification 49.18%, LOH 28.14%, copy-neutral LOH 19.86%, and homozygous deletion 1.11%) with mean 1.68% (range: 1.03%–2.06%) of the genome being heterozygous diploid, consistent with previous reports (15, 29). Patient specific details are shown in the Supplementary Data. Allele specific copy-number analysis identified a mean aberrant cell fraction of 0.79 in our samples suggesting high tumor content for all samples assayed, with a mean ploidy of 2.6 (Table 2; Supplementary Fig. S4).) which is consistent with previous reports (30).

**TABLE 2:** Ploidy and aberrant cell fraction in samples

Sample	02_Rec1	02_Rec2	02_Rec3	04_Rec1	04_Rec2	07_Rec1	07_Rec2	07_Rec3
Aberrant cell fraction	0.72	0.75	0.85	0.91	0.73	0.78	0.89	0.75
Ploidy	2.638999	2.621263	3.727031	2.481872	2.28313	2.242088	2.610261	2.505003

All 3 patients showed copy-number losses at sites of known tumor suppressor genes and gains at the sites of known oncogenes consistent with previous reports (Supplementary Fig. S5). The complete list of gene-specific copy number in 3 patients is listed in Supplementary Table S4.

### TNBC Shows Branching Pattern of Clonal Evolution with a Persistent Stem Clone

**FIGURE 2:**
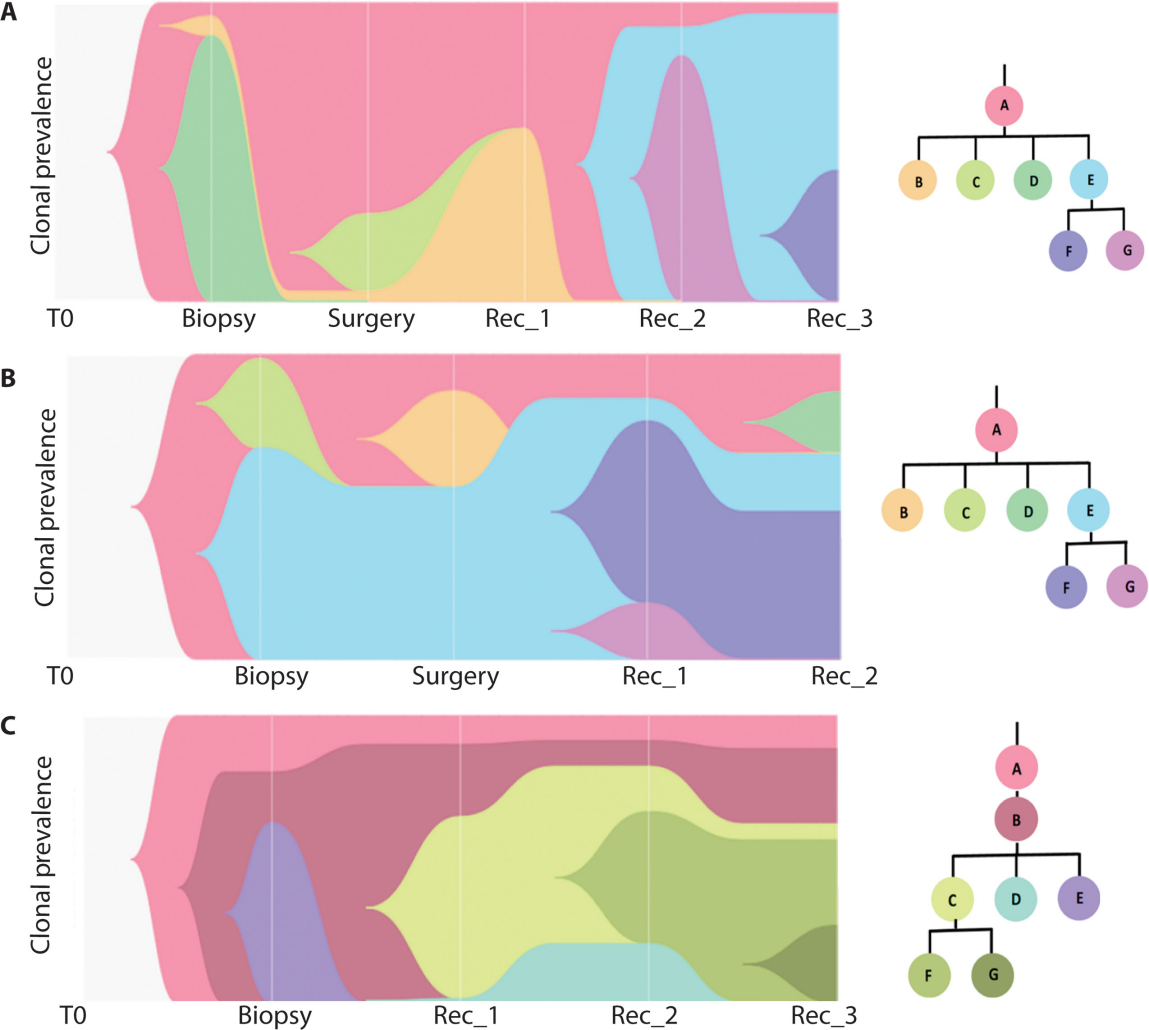
Graphical representation of clonal evolution observed in sequentially collected samples over time in 3 patients with TNBC. Timepoints are depicted on the X-axis and Cellular Prevalence (Clonal Prevalence) is shown on the Y-axis. Different colors in each patient represent various clones and their changing dynamics over time. Clonal phylogeny for each patient is shown on the left side of main figure. **A,** Patient_02. **B,** Patient_04. **C,** Patient_07.

Phylogenetic clonal evolution in the 3 patients is shown in Fig. 2. The tumor comprised a founder clone and two subclones in all 3 patients at the time of diagnosis. Tumors from all 3 patients in our study exhibited a branching pattern of evolution, with one stem clone persisting through the lifespan of cancer, giving rise to various subclones by acquiring additional mutations, which in turn propagated further subclones of their own (Fig. 2). The stem clone (clone A, Fig. 2) in all 3 patients contained at least one Tier 1 mutation, that is, a known cancer driver mutation. However, only a few subclones gained additional Tier 1 mutations which were absent in the stem clone.

#### Patient_02

In Patient_02 (germline *BRCA1* mutated), we identified seven clones (one stem and six subclones) comprised of 311 mutations in five samples collected sequentially (Fig. 2A). The treatment-naïve primary tumor biopsy obtained at diagnosis (02_Bio) showed three clones: stem clone A (cellular prevalence = 0.01), and subclones B (CP = 0.02) and D (CP = 0.21). Stem clone A which persisted through the disease course contained two Tier 1 mutations (*PDGFRB* p.V761I, *COL3A1* p.S1425I), four Tier 2 mutations (*ARID2* p.S564X), and 11 Tier 3 mutations (*NECTIN3* p.T226M, *MYH13* p.N1922S). Subclones C, E, F, and G were gained at subsequent timepoints, of which C and F were lost but E and G persisted up to the point of last biopsy. One of the initial subclones, B, was also lost in the last biopsy. The complete list of mutations with their functional annotation is provided in the Supplementary Table S5 and detailed description of clonal architecture and dynamics over time are explained in Supplementary Data.

#### Patient_04

In Patient_04, we identified 518 total mutations in four sequentially collected samples, which were classified into 19 clones by PyClone, of which nine clones were comprised of a single mutation each (eight Tier 4 mutations and one Tier 2 mutation). We excluded these nine clones from further phylogenetic analysis and visualization using CALDER. The complete list of mutations with their functional annotation is provided in Supplementary Table S6. Three of the 10 clones (named clone AA, BB, and CC) could not be visualized by CALDER because very high read counts led to narrow confidence intervals and the inability to find values common to all clusters. Widening the confidence interval did not yield an optimum tree presenting all 10 clones. Therefore, these three clones comprising of 66 mutations (clone AA 48 mutations, clone BB 4 mutations, clone CC 14 mutations) are not represented in the clonality analysis. Of note, 62 of 66 mutations in these three clones were Tier 4 mutations. We present the PyClone analysis and CALDER visualization for seven clones from four samples collected sequential in this patient (Fig. 2B). The treatment-naïve primary tumor biopsy (04_Bio) showed three clones—stem clone A with cellular prevalence 0.01 (18 mutations), subclone C with cellular prevalence 0.21 (27 mutations), and subclone E with cellular prevalence 0.49 (four mutations). Subclones B, D, F, and G were gained at subsequent timepoints, of which B and G were lost but D and F persisted up to the point of last biopsy. One of the initial subclones, E persisted but C was lost in the last biopsy. Stem Clone A, which contained one Tier 1 mutation (*TP53* p.I119S), two Tier 2 mutations (*BRAF* p.Q386 L and *KMT2C* p.G4125C), and two Tier 3 mutations (*GOLGB1* p.P2841 L *KDM4E* p.R240W), underwent clonal expansion with cellular prevalence 0.06 and persisted up to the last biopsy. Detailed description of clonal architecture and dynamics over time are explained in Supplementary Data.

#### Patient_07

In Patient_07, we identified seven clones comprised of 142 mutations in four samples collected during the disease course (Fig. 2C). The complete list of mutations with their functional annotation is provided in Supplementary Table S7. Treatment-naïve primary tumor biopsy (07_Bio) showed three clones—stem clone A with a cellular prevalence of 0.15, subclone B with a cellular prevalence of 0.13, and subclone E with a cellular prevalence of 0.49. This patient showed a unique pattern of clonal evolution wherein stem clone A resulted in a daughter subclone B from the very beginning, and subclone B persisted throughout the tumor's life course, giving rise to all other subclones. Stem clone A, which persisted throughout, contained one Tier 1 mutation (*TP53* p.R81X), seven Tier 2 mutations (*APC* p.S1393T, *ESR1* p.F591L, *EZH2* p.D36N, *FAT3* p.K251N), and four Tier 3 mutations (*SCN5A* p.V1759M). Subclone B gained four Tier 4 mutations but did not gain any Tier 1, 2, or 3 mutations, while subclone E gained one Tier 2 mutation (*ZEB1* p.K419I) and three Tier 3 mutations. Subclones E, C, D, F, and G were gained at subsequent timepoints, of which E and D were lost but C, F, and G were persisted up to the point of last biopsy. One of the initial subclones, E, persisted but C was lost in the last biopsy. Detailed description of clonal architecture and dynamics over time are explained in Supplementary Data.

### ctDNA Captures Clonal and Subclonal Mutations with Higher Sensitivity Compared with Tissue Biopsy

**FIGURE 3:**
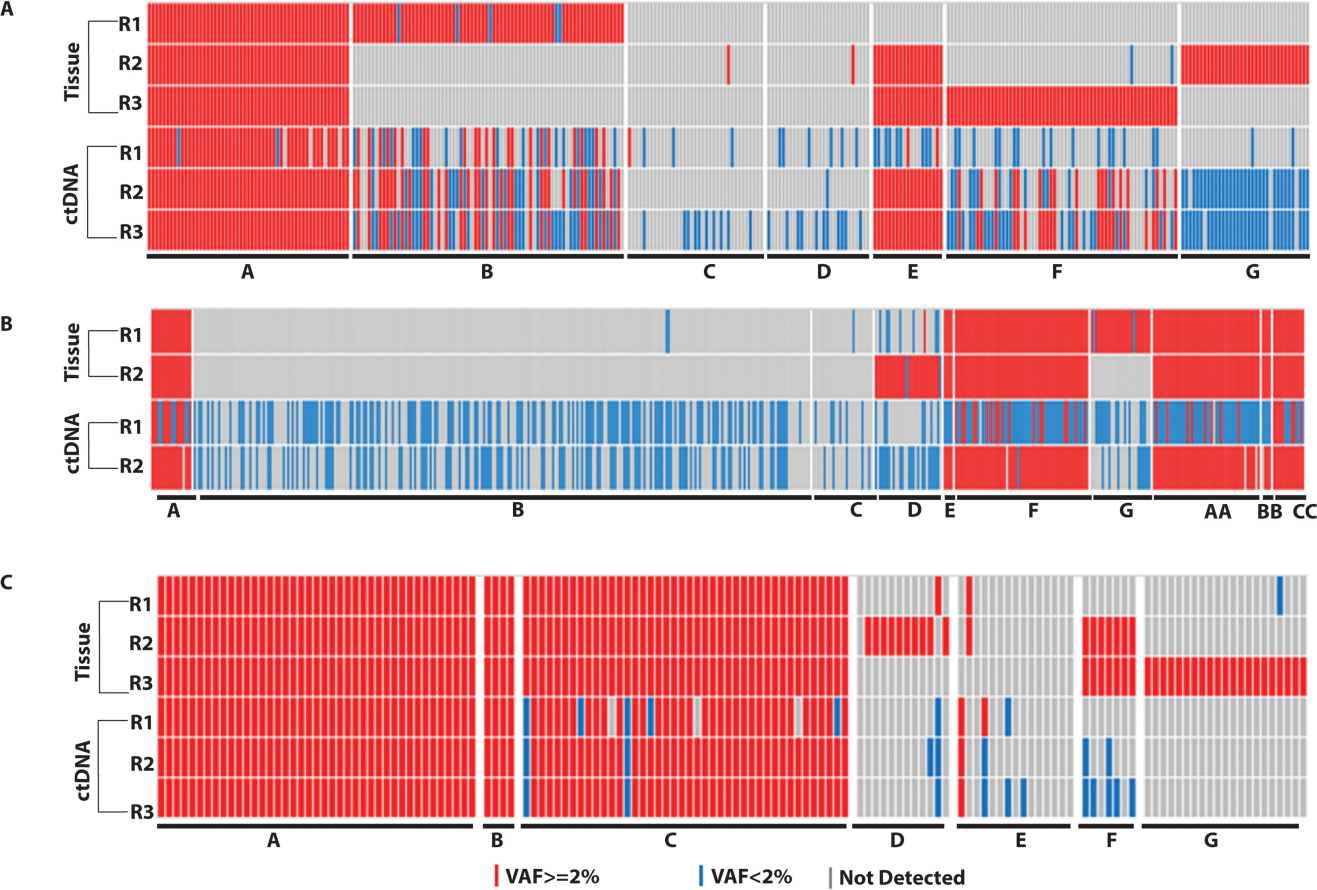
Heat map representation of mutations identified in fresh-frozen recurrent tumor samples and corresponding plasma samples. Mutations were clustered on the basis of clonal structure identified from Pyclone and renamed as in CALDER tool. Red rectangles denote high confidence mutations with variant allele frequency (VAF) > 2%, while blue rectangles indicate mutations with statistical significance but VAF < 2%. **A,** Patient_02. **B,** Patient_04. Last three clusters (AA, BB, and CC) were identified in Pyclone analysis but not represented in CALDER. **C,** Patient_07.

Deep sequencing of custom panel of somatic mutations was performed on eight plasma-derived ctDNA samples collected at the time of disease progression in the 3 patients, at an average coverage of 30,000X. In all 3 patients, all mutations identified in the stem clones were also present in ctDNA from respective plasma samples with a prevalence of more than 2% variant allele frequency (Fig. 3), as also reported by others (31). Furthermore, some subclones which could not be detected in high-depth tissue sequencing at some timepoints, were detected with low variant allele frequency in corresponding ctDNA as shown in Fig. 3 and described in detail in Supplementary Data. This suggests that ctDNA analysis can potentially better evaluate subclonal architecture compared with single-site tissue biopsy.

### Many Stem Clone Mutations Continue to be Expressed During Tumor Evolution

**FIGURE 4:**
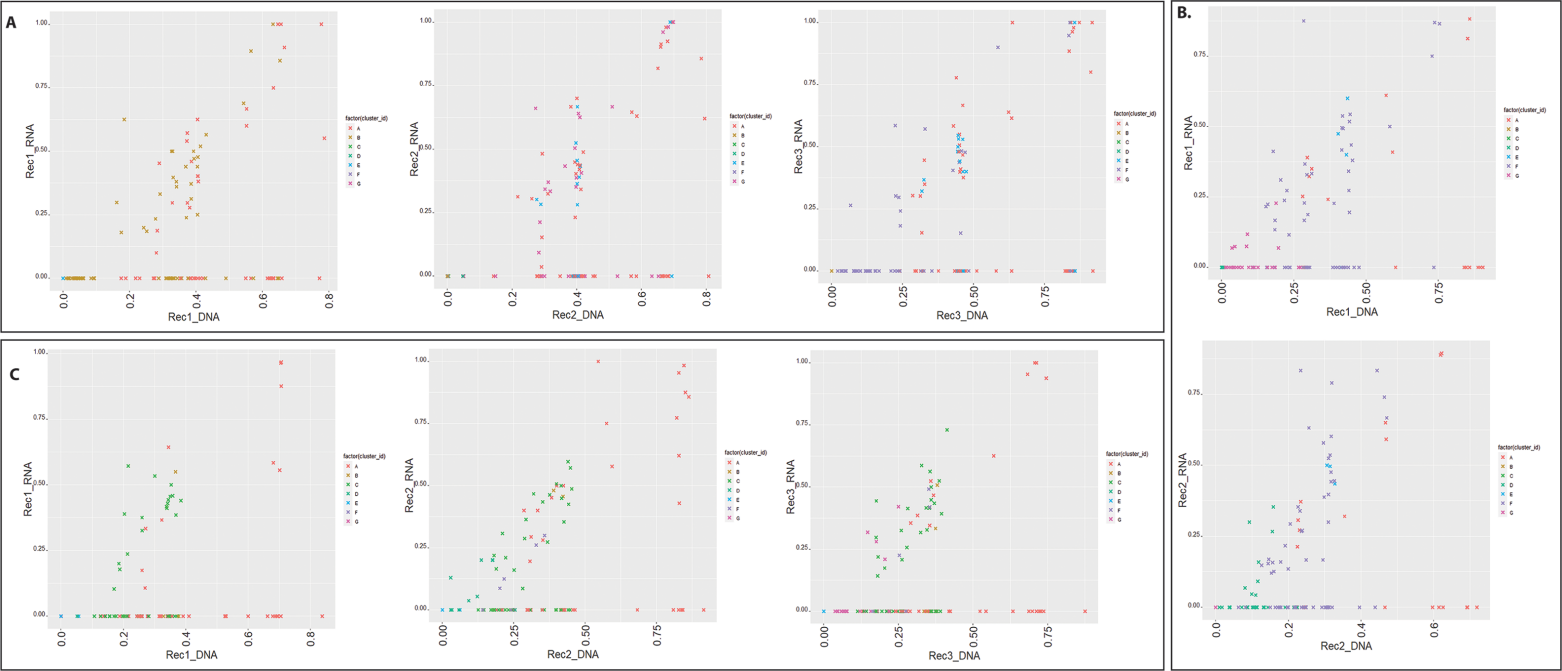
Sample-specific allele frequencies from deep sequencing were compared with allele-specific gene expression frequency from RNA-seq experiment. Colors indicate individual clones identified from PyClone, and each mutation is represented by “×” symbol. **A,** Patient_02. **B,** Patient_04. **C,** Patient_07.

Variant allele frequencies were inferred from RNA-seq of eight sequential samples in 3 patients. Our results suggest that many somatic mutations, including stem and branch mutations, were expressed at the RNA level. In five of eight samples, stem clone mutations were the sole highest expressed variants and in the remaining three samples both stem and some non-stem mutations were present among the highest expressed variants. These results are shown in Fig. 4 and described in detail in Supplementary Data.

## Discussion

We report the clonal architecture and evolution pattern in 3 patients with metastatic TNBC, 2 of whom had germline pathogenic *BRCA1* mutation (Supplementary Table S8), using a sequential, multiple timepoint, multiomics analysis. Notably, the sample set in all 3 patients comprised the initial diagnostic biopsy in treatment-naïve non-metastatic stage, through to the ultimately fatal metastatic tumor, with intervening biopsies at each progression. Our analysis suggests that the primary non-metastatic tumor was polyclonal in all patients, comprising a stem clone and daughter clones. The tumor remained polyclonal throughout its life history with persistence of the stem clone. However, daughter clones were extinguished and gained at various timepoints, likely under the selective pressure of various chemotherapy agents (Supplementary Table S9). Interestingly, this pattern was similar in 2 patients with germline *BRCA1* mutation and the single patient without any germline pathogenic abnormality. There was no reversion of the *BRCA1* mutation in somatic tumor tissue of both patients throughout their clinical course. Our analysis suggests that clonal biology and evolution are likely to be similar in those with and without germline predisposition (32). Our analysis also suggests that the subsequent relapses in 2 patients with germline *BRCA1* mutation were derived from the primary index tumor and were not second cancers. Transcriptome data from all samples in all patients confirmed their classification into the basal-like category, irrespective of the physical and temporal distance from the primary tumor, attesting to an enduring intrinsic subtype pattern through several relapses in patients with TNBC.

We detected the branching evolution pattern of mutations through the disease course in all 3 patients, with a stem clone comprising stem mutations, which acquired further mutations to branch into daughter clones. Because the stem clone was present throughout the clinical course in all 3 patients, the mutations comprising these clones could be related to treatment resistance. Notably, many mutations comprising the stem clone continued to be expressed in multiple timepoint transcriptomic analysis, further suggesting their possible association with treatment resistance. The acquisition and extinction of subclones suggest the possibility of their correlation with treatment resistance and sensitivity, respectively. Some daughter clones in initial biopsy and subsequent surgery were extinguished during metastatic evolution, suggesting that these were associated with sensitivity to (neo) adjuvant chemotherapy and radiotherapy.

In line with previous reports (4, 14, 23, 33), there was minimal overlap in the mutational landscape across the 3 patients, with only *TP53* (different hotspot mutations in the 3 patients) being the commonly mutated gene. These findings affirm the marked intertumor heterogeneity in TNBC, a potential challenge for the personalized medicine paradigm in these patients.

We found enrichment of signature 3 [homologous recombination deficiency (HRD) signature] in the 3 patients, persisting in all sequential samples, including in 1 patient without germline pathogenic *BRCA1* mutation. This may have implications for treating patients with HRD-positive TNBC with DNA-damaging agents such as PARP inhibitors. Signature 3 has been previously reported in breast and pancreatic cancers and shows a strong association with germline and somatic *BRCA1* and *BRCA2* mutations, leading to defective homologous recombination repair of DNA double-strand breaks (34–36). This is consistent with the presence of germline *BRCA1* mutations in 2 of our patients, while the presence of signature 3 in the third patient's tumor could be due to epigenetic silencing of homologous recombination repair genes. We also found signature 13 in all 3 patients in tumor samples obtained at 11 of 13 timepoints. This signature is associated with the AID/APOBEC family of cytidine deaminases (35), has been reported to be widespread in human cancers (37), is usually associated with HER2-like tumors (38), but has also been reported in TNBC (39). We found germline polymorphisms in APOBEC family genes in 2 of the 3 patients, one of whom also had hepatitis C, which has also been associated with the presence of APOBEC signature in tumors (37, 40, 41). Others have also reported the co-occurrence of signatures 3 and 13 in breast cancer (42). The presence of APOBEC signature may be associated with a benefit of immunotherapy (43), and we can speculate about the possible benefit of the combination of immunotherapy and PARP inhibitors in patients with such tumors. Our data suggest that mutational signatures that predominate at the time of diagnosis remain stable and continue to be the predominant signatures throughout the life course of a tumor through several exposures to different chemotherapy agents. Our data corroborate the results of Nik-Zainal and colleagues (16) who subjected 20 primary breast cancer samples to a 30–40X sequencing depth and applied a novel common ancestor statistical approach to identify evolutionary periods underlying subclonal divergence. Their data suggest that the accumulation of thousands of mutations is required for subclones to emerge, suggesting that cancer-specific signatures of point mutations and genomic instability emerge at late-stage disease. A limitation of their study was a lack of sequential samples from the same patients over time, which we could accomplish in this study.

The median tumor mutation burden (TMB) in our sample set was 1.59 mutations/Mb, which is consistent with previous reports in TNBC (4). Notably, TMB did not markedly change between non-metastatic and metastatic tumor samples in our patients.

We performed SNP arrays to infer copy numbers for eight fresh samples from recurrent/metastatic sites in 3 patients, in whom the mean ploidy was 2.63, consistent with previous reports (44). We identified a high level of copy-number changes in our patients attesting to the high genomic instability in metastatic TNBC, which has been reported earlier (39, 44–46). Gao and colleagues (15) subjected 1,000 single cells from 12 patients with TNBC at diagnosis and found that TNBCs exhibited punctuated copy-number evolution, with a clonal blast occurring at presentation with copy numbers remaining stable throughout the disease. Each patient had one to three major clonal subpopulations that shared a common evolutionary lineage. More recently, the same group has reported (47) that most cancer cells undergo a period of transient instability wherein a large number of subclones are produced, followed by steady ongoing copy-number evolution that persists during clonal expansion of the tumor. This led the group to propose a revised model of copy-number evolution in TNBC under which TP53 mutations occur early, resulting in genomic instability with acquisition of subclones which continue to evolve as the tumor expands. In line with this finding, we observed early acquisition of TP53 mutations in all 3 patients, a high number of copy-number gains and losses in many cancer-related and other genes in these samples, followed by transient instability, with discordant copy-number profiles in 40%, 22.54%, and 40.21% reduced segments, respectively, in sequential samples from the 3 patients. Our findings of a substantial concordance of copy-number profiles in the evolving tumor suggest that CNAs could constitute stable targets for treatment throughout the life course of these tumors, should one or more of these aberrations be proven to be the drivers.

Methodologically, we found a strong correlation between WES and ultra-high depth targeted sequencing for variant allele frequency of each mutation (*R* > 0.9 for all samples; Supplementary Fig. S2), which has also been reported by others (48). This suggests that WES at about 200X depth may be adequate to infer the clonal architecture of tumors. Ultra-high depth sequencing captured some additional subclonal mutations present at very low allelic prevalence but given the high correlation of VAF between the two techniques, it is unlikely that clonal architecture would differ if derived from WES compared with deep sequencing data.

In line with previous findings (31), our deep sequencing analysis (∼30,000X) of ctDNA can recapitulate the clonal architecture of tumors at all timepoints. We detected the stem clone mutations in ctDNA from corresponding plasma samples with high confidence and more than 2% VAF (Fig. 3). In addition, we could detect some mutations in ctDNA which were missed in tumor biopsy, likely because of intratumor heterogeneity of the biopsied lesion. Another possibility is that these clones were seeded from a metastatic site that we did not biopsy, and a remote possibility is that these clones were seeded from a tumor that was as yet clinically undetected.

Other, single timepoint analyses (49), have suggested a simple clonal organization comprising a stem and daughter clone(s). This can be resolved into more detailed clonal architecture only when samples from other timepoints are available, which show differing variant allele frequencies of various subclones. Kim and colleagues (18) in their single-cell analysis of TNBC before and after neoadjuvant chemotherapy described clonal persistence or extinction in response to treatment, indicating that resistance occurred as a result of adaptive selection of genomic aberrations present at the time of diagnosis. When the detailed clonal architecture of the evolving tumor is available at various timepoints along with treatments that preceded each sample, as is the case in our dataset, it may be possible to find associations between the acquisition and extinction of clones and treatment sensitivity and resistance. In contrast, the multiple-location, single-timepoint sampling can only demonstrate spatial heterogeneity.

Our analysis showed an interesting finding in 1 patient (P2) in whom subclone B was present in initial treatment-naïve biopsy, slightly expanded in the post-neoadjuvant surgical specimen, present in the first-relapse lung metastasis sample, present in ctDNA at first relapse and two subsequent disease progressions, but absent from local chest wall samples at progressions 2 and 3. This suggests that subclones can already be seeded in distant metastatic sites at the initial diagnosis, may have tropism for particular organs, and that ctDNA may provide a complementary and perhaps more complete picture of the entire tumor burden in patients with multiple sites of metastases. Previous studies in patients with TNBC similarly suggest that different clones pre-existing in the primary tumor subsequently seed multiple metastatic lesions (22, 50). Although we biopsied only one tumor site at progression, the fact that these changes were captured in the ctDNA at all progression timepoints, suggests that our findings are in line with previous reports.

The ctDNA sampling, multiomics analytic strategy, and inclusion of patients with germline pathogenic *BRCA1* mutations are other important aspects of our study. Although there is no strong *a priori* reason for TNBC tumor evolution to differ in patients with and without gBRCA pathogenic variants, empirical proof of clonal patterns is provided by our study.

The patients in this study had a rapid clinical deterioration after relapse. However, after relapse, the median overall survival of patients with TNBC is known to be short, in the range of 12–15 months. They eventually, and relatively rapidly, became refractory to treatments after experiencing relapse, which is consistent with the biological behavior of relapsed TNBC. Given the short clinical course of all 3 patients, it is possible that inherent tumor evolutionary mechanisms and chemotherapy-related genotoxicity could have contributed to the branching pattern seen in our study. The design of our study does not allow evaluation of the contributing mechanisms.

The main limitation of our study is its small sample size of 3 patients. Therefore, our findings, including branching evolution pattern of mutations, will have to be considered as preliminary and need to be replicated in larger cohorts. However, it needs to be appreciated that repeated sequential tumor sampling from individual patients through several episodes of disease progression is a challenging sample set to assemble. Second, we did not obtain samples from tumor after death, so additional terminal-stage spatial heterogeneity was possibly not captured, especially from metastatic sites such as the brain, which are difficult to access. Therefore, our findings need to be replicated in larger cohorts, including autopsy-based samples. Third, each patient's initial diagnostic biopsy and surgical specimen tissue were retrospectively obtained as paraffin-embedded blocks. Although the pathologist selected the most suitable block for inclusion, it is possible that the quality of extracted DNA extracted from some of this retrospectively collected material might have been lower. This could have led to systematically different tumor mutational burden between fresh tissue from metastatic sites and archival FFPE tissue.

In summary, our analysis of a sequentially sampled TNBC patient cohort suggests the presence of branching evolutionary pattern of mutations, widespread copy-number aberrations, stability of mutational signatures and intrinsic subtype over disease course, high interpatient tumor heterogeneity, and the ability of ultra-high depth sequenced ctDNA to recapitulate the clonal architecture of the somatic tumor. The evolutionary pattern was similar in patients with and without germline pathogenic *BRCA1* mutations.

## Supplementary Material

Supplementary Data This file contains supplementary data for this study.

Supplementary Materials and Methods This file contains the supplementary materials and methods for this study.

Supplementary figure S1 This figure shows peaks from Sanger sequencing of germline variants identified in two patient samples.

Supplementary figure S2 This figure describes Co-relation of VAFs for deep-sequencing of Whole Exome Sequencing data from tumour samples.

Supplementary figure S3 This figure shows the distribution of point mutations identified in WES and ultra-deep Sequencing.

Supplementary figure S4 This figure shows ASCAT copy number profile of each sample.

Supplementary figure S5 This figure shows the SGOL score for all patient samples with clinically relevant genes.

Supplementary Table S1 This table shows the relevant clinical and assay information for patients on this study.

Supplementary Table S2 This table lists all the mutations that have been identified in whole exome sequencing data from all patient samples

Supplementary Table S3 This table shows the primer information for all point mutations in the targetted ultra-deep sequencing assay.

Supplementary Table S4 This tables shows the copy number variation data for all patients on this study

Supplementary Table S5 This table shows the annotation information from Annovar for clonal architecture of Patient P2

Supplementary Table S6 This table shows the annotation information from Annovar for clonal architecture of Patient P4

Supplementary Table S7 This table shows the annotation information from Annovar for clonal architecture of Patient P7

Supplementary Table S8 This table shows the genes in the germline sequencing panel for the targetted germline mutation assay.

Supplementary Table S9 This table lists the cellular prevalence values for clones across each patient on this study.

## References

[1] Liedtke C , Mazouni C, Hess KR, Andre F, Tordai A, Mejia JA, . Response to neoadjuvant therapy and long-term survival in patients with triple-negative breast cancer. J Clin Oncol 2008;26:1275–81. 18250347 10.1200/JCO.2007.14.4147

[2] Gupta S . Triple negative breast cancer: a continuing challenge. Indian J Med Paediatr Oncol 2013;34:1–2. 23878477 10.4103/0971-5851.113393 PMC3715970

[3] Parker JS , Mullins M, Cheang MC, Leung S, Voduc D, Vickery T, . Supervised risk predictor of breast cancer based on intrinsic subtypes. J Clin Oncol 2009;27:1160–7. 19204204 10.1200/JCO.2008.18.1370 PMC2667820

[4] Cancer Genome Atlas Network. Comprehensive molecular portraits of human breast tumours. Nature 2012;490:61–70. 23000897 10.1038/nature11412 PMC3465532

[5] Ciriello G , Gatza ML, Beck AH, Wilkerson MD, Rhie SK, Pastore A, . Comprehensive molecular portraits of invasive lobular breast cancer. Cell 2015;163:506–19. 26451490 10.1016/j.cell.2015.09.033 PMC4603750

[6] Nik-Zainal S , Davies H, Staaf J, Ramakrishna M, Glodzik D, Zou X, . Landscape of somatic mutations in 560 breast cancer whole-genome sequences. Nature 2016;534:47–54. 27135926 10.1038/nature17676 PMC4910866

[7] Berger AC , Korkut A, Kanchi RS, Hegde AM, Lenoir W, Liu W, . A comprehensive pan-cancer molecular study of gynecologic and breast cancers. Cancer Cell 2018;33:690–705. 29622464 10.1016/j.ccell.2018.03.014 PMC5959730

[8] Lehmann BD , Bauer JA, Chen X, Sanders ME, Chakravarthy AB, Shyr Y, . Identification of human triple-negative breast cancer subtypes and preclinical models for selection of targeted therapies. J Clin Invest 2011;121:2750–67. 21633166 10.1172/JCI45014 PMC3127435

[9] Bareche Y , Venet D, Ignatiadis M, Aftimos P, Piccart M, Rothe F, . Unravelling triple-negative breast cancer molecular heterogeneity using an integrative multiomic analysis. Ann Oncol 2018;29:895–902. 29365031 10.1093/annonc/mdy024 PMC5913636

[10] Jezequel P , Kerdraon O, Hondermarck H, Guerin-Charbonnel C, Lasla H, Gouraud W, . Identification of three subtypes of triple-negative breast cancer with potential therapeutic implications. Breast Cancer Res 2019;21:65. 31101122 10.1186/s13058-019-1148-6 PMC6525459

[11] Wang DY , Jiang Z, Ben-David Y, Woodgett JR, Zacksenhaus E. Molecular stratification within triple-negative breast cancer subtypes. Sci Rep 2019;9:19107. 31836816 10.1038/s41598-019-55710-w PMC6911070

[12] Funakoshi Y , Wang Y, Semba T, Masuda H, Hout D, Ueno NT, . Comparison of molecular profile in triple-negative inflammatory and non-inflammatory breast cancer not of mesenchymal stem-like subtype. PLoS One 2019;14:e0222336. 31532791 10.1371/journal.pone.0222336 PMC6750603

[13] Lehmann BD , Jovanovic B, Chen X, Estrada MV, Johnson KN, Shyr Y, . Refinement of triple-negative breast cancer molecular subtypes: implications for neoadjuvant chemotherapy selection. PLoS One 2016;11:e0157368. 27310713 10.1371/journal.pone.0157368 PMC4911051

[14] Shah SP , Roth A, Goya R, Oloumi A, Ha G, Zhao Y, . The clonal and mutational evolution spectrum of primary triple-negative breast cancers. Nature 2012;486:395–9. 22495314 10.1038/nature10933 PMC3863681

[15] Gao R , Davis A, McDonald TO, Sei E, Shi X, Wang Y, . Punctuated copy number evolution and clonal stasis in triple-negative breast cancer. Nat Genet 2016;48:1119–30. 27526321 10.1038/ng.3641 PMC5042845

[16] Nik-Zainal S , Van Loo P, Wedge DC, Alexandrov LB, Greenman CD, Lau KW, . The life history of 21 breast cancers. Cell 2012;149:994–1007. 22608083 10.1016/j.cell.2012.04.023 PMC3428864

[17] Yates LR , Gerstung M, Knappskog S, Desmedt C, Gundem G, Van Loo P, . Subclonal diversification of primary breast cancer revealed by multiregion sequencing. Nat Med 2015;21:751–9. 26099045 10.1038/nm.3886 PMC4500826

[18] Kim C , Gao R, Sei E, Brandt R, Hartman J, Hatschek T, . Chemoresistance evolution in triple-negative breast cancer delineated by single-cell sequencing. Cell 2018;173:879–93. 29681456 10.1016/j.cell.2018.03.041 PMC6132060

[19] Savage P , Pacis A, Kuasne H, Liu L, Lai D, Wan A, . Chemogenomic profiling of breast cancer patient-derived xenografts reveals targetable vulnerabilities for difficult-to-treat tumors. Commun Biol 2020;3:310. 32546838 10.1038/s42003-020-1042-x PMC7298048

[20] Salehi S , Kabeer F, Ceglia N, Andronescu M, Williams MJ, Campbell KR, . Clonal fitness inferred from time-series modelling of single-cell cancer genomes. Nature 2021;595:585–90. 34163070 10.1038/s41586-021-03648-3 PMC8396073

[21] Echeverria GV , Ge Z, Seth S, Zhang X, Jeter-Jones S, Zhou X, . Resistance to neoadjuvant chemotherapy in triple-negative breast cancer mediated by a reversible drug-tolerant state. Sci Transl Med 2019;11:eaav0936. 30996079 10.1126/scitranslmed.aav0936 PMC6541393

[22] Echeverria GV , Powell E, Seth S, Ge Z, Carugo A, Bristow C, . High-resolution clonal mapping of multi-organ metastasis in triple negative breast cancer. Nat Commun 2018;9:5079. 30498242 10.1038/s41467-018-07406-4 PMC6265294

[23] Hoadley KA , Siegel MB, Kanchi KL, Miller CA, Ding L, Zhao W, . Tumor evolution in two patients with basal-like breast cancer: a retrospective genomics study of multiple metastases. PLoS Med 2016;13:e1002174. 27923045 10.1371/journal.pmed.1002174 PMC5140046

[24] Savas P , Teo ZL, Lefevre C, Flensburg C, Caramia F, Alsop K, . The subclonal architecture of metastatic breast cancer: results from a prospective community-based rapid autopsy program “CASCADE”. PLoS Med 2016;13:e1002204. 28027312 10.1371/journal.pmed.1002204 PMC5189956

[25] Gupta S , Rajappa S, Advani S, Agarwal A, Aggarwal S, Goswami C, . Prevalence of BRCA1 and BRCA2 mutations among patients with ovarian, primary peritoneal, and fallopian tube cancer in India: a multicenter cross-sectional study. JCO Glob Oncol 2021;7:849–61. 34101484 10.1200/GO.21.00051 PMC8457852

[26] Roth A , Khattra J, Yap D, Wan A, Laks E, Biele J, . PyClone: statistical inference of clonal population structure in cancer. Nat Methods 2014;11:396–8. 24633410 10.1038/nmeth.2883 PMC4864026

[27] Myers MA , Satas G, Raphael BJ. CALDER: inferring phylogenetic trees from longitudinal tumor samples. Cell Syst 2019;8:514–22. 31229560 10.1016/j.cels.2019.05.010 PMC7263382

[28] Smith MA , Nielsen CB, Chan FC, McPherson A, Roth A, Farahani H, . E-scape: interactive visualization of single-cell phylogenetics and cancer evolution. Nat Methods 2017;14:549–50. 28557980 10.1038/nmeth.4303

[29] Turner N , Lambros MB, Horlings HM, Pearson A, Sharpe R, Natrajan R, . Integrative molecular profiling of triple negative breast cancers identifies amplicon drivers and potential therapeutic targets. Oncogene 2010;29:2013–23. 20101236 10.1038/onc.2009.489 PMC2852518

[30] Jiang YZ , Ma D, Suo C, Shi J, Xue M, Hu X, . Genomic and transcriptomic landscape of triple-negative breast cancers: subtypes and treatment strategies. Cancer Cell 2019;35:428–40. 30853353 10.1016/j.ccell.2019.02.001

[31] Murtaza M , Dawson SJ, Pogrebniak K, Rueda OM, Provenzano E, Grant J, . Multifocal clonal evolution characterized using circulating tumour DNA in a case of metastatic breast cancer. Nat Commun 2015;6:8760. 26530965 10.1038/ncomms9760 PMC4659935

[32] Yates LR , Knappskog S, Wedge D, Farmery JHR, Gonzalez S, Martincorena I, . Genomic evolution of breast cancer metastasis and relapse. Cancer Cell 2017;32:169–84. 28810143 10.1016/j.ccell.2017.07.005 PMC5559645

[33] Garcia-Recio S , Hinoue T, Wheeler GL, Kelly BJ, Garrido-Castro AC, Pascual T, . Multiomics in primary and metastatic breast tumors from the AURORA US network finds microenvironment and epigenetic drivers of metastasis. Nat Cancer 2023;4:128–47. 36585450 10.1038/s43018-022-00491-x PMC9886551

[34] Alexandrov LB , Nik-Zainal S, Wedge DC, Aparicio SA, Behjati S, Biankin AV, . Signatures of mutational processes in human cancer. Nature 2013;500:415–21. 23945592 10.1038/nature12477 PMC3776390

[35] Alexandrov LB , Kim J, Haradhvala NJ, Huang MN, Tian Ng AW, Wu Y, . The repertoire of mutational signatures in human cancer. Nature 2020;578:94–101. 32025018 10.1038/s41586-020-1943-3 PMC7054213

[36] Watkins J , Weekes D, Shah V, Gazinska P, Joshi S, Sidhu B, . Genomic complexity profiling reveals that HORMAD1 overexpression contributes to homologous recombination deficiency in triple-negative breast cancers. Cancer Discov 2015;5:488–505. 25770156 10.1158/2159-8290.CD-14-1092 PMC4490184

[37] Roberts SA , Lawrence MS, Klimczak LJ, Grimm SA, Fargo D, Stojanov P, . An APOBEC cytidine deaminase mutagenesis pattern is widespread in human cancers. Nat Genet 2013;45:970–6. 23852170 10.1038/ng.2702 PMC3789062

[38] Angus L , Smid M, Wilting SM, van Riet J, Van Hoeck A, Nguyen L, . The genomic landscape of metastatic breast cancer highlights changes in mutation and signature frequencies. Nat Genet 2019;51:1450–8. 31570896 10.1038/s41588-019-0507-7 PMC6858873

[39] Kawazu M , Kojima S, Ueno T, Totoki Y, Nakamura H, Kunita A, . Integrative analysis of genomic alterations in triple-negative breast cancer in association with homologous recombination deficiency. PLos Genet 2017;13:e1006853. 28636652 10.1371/journal.pgen.1006853 PMC5500377

[40] Zhu YP , Peng ZG, Wu ZY, Li JR, Huang MH, Si SY, . Host APOBEC3G protein inhibits HCV replication through direct binding at NS3. PLoS One 2015;10:e0121608. 25811715 10.1371/journal.pone.0121608 PMC4374698

[41] Nik-Zainal S , Alexandrov LB, Wedge DC, Van Loo P, Greenman CD, Raine K, . Mutational processes molding the genomes of 21 breast cancers. Cell 2012;149:979–93. 22608084 10.1016/j.cell.2012.04.024 PMC3414841

[42] Denkert C , Untch M, Benz S, Schneeweiss A, Weber KE, Schmatloch S, . Reconstructing tumor history in breast cancer: signatures of mutational processes and response to neoadjuvant chemotherapy(small star, filled). Ann Oncol 2021;32:500–11. 33418062 10.1016/j.annonc.2020.12.016

[43] Wang S , Jia M, He Z, Liu XS. APOBEC3B and APOBEC mutational signature as potential predictive markers for immunotherapy response in non-small cell lung cancer. Oncogene 2018;37:3924–36. 29695832 10.1038/s41388-018-0245-9 PMC6053356

[44] Van Loo P , Nordgard SH, Lingjaerde OC, Russnes HG, Rye IH, Sun W, . Allele-specific copy number analysis of tumors. Proc Nat Acad Sci U S A 2010;107:16910–5. 10.1073/pnas.1009843107 PMC2947907 20837533

[45] Hancock BA , Chen YH, Solzak JP, Ahmad MN, Wedge DC, Brinza D, . Profiling molecular regulators of recurrence in chemorefractory triple-negative breast cancers. Breast Cancer Res 2019;21:87. 31383035 10.1186/s13058-019-1171-7 PMC6683504

[46] Gao R , Bai S, Henderson YC, Lin Y, Schalck A, Yan Y, . Delineating copy number and clonal substructure in human tumors from single-cell transcriptomes. Nat Biotechnol 2021;39:599–608. 33462507 10.1038/s41587-020-00795-2 PMC8122019

[47] Minussi DC , Nicholson MD, Ye H, Davis A, Wang K, Baker T, . Breast tumours maintain a reservoir of subclonal diversity during expansion. Nature 2021;592:302–8. 33762732 10.1038/s41586-021-03357-x PMC8049101

[48] Weber ZT , Collier KA, Tallman D, Forman J, Shukla S, Asad S, . Modeling clonal structure over narrow time frames via circulating tumor DNA in metastatic breast cancer. Genome Med 2021;13:89. 34016182 10.1186/s13073-021-00895-x PMC8136103

[49] Miller CA , White BS, Dees ND, Griffith M, Welch JS, Griffith OL, . SciClone: inferring clonal architecture and tracking the spatial and temporal patterns of tumor evolution. PLoS Comput Biol 2014;10:e1003665. 25102416 10.1371/journal.pcbi.1003665 PMC4125065

[50] Brown D , Smeets D, Szekely B, Larsimont D, Szasz AM, Adnet PY, . Phylogenetic analysis of metastatic progression in breast cancer using somatic mutations and copy number aberrations. Nat Commun 2017;8:14944. 28429735 10.1038/ncomms14944 PMC5474888

