# Supplementary material for: Natural History of Germline BRCA1 Mutated and BRCA Wild-type Triple-negative Breast Cancer
Source: Cancer Res Commun. 2024 Feb 14;4(2):404–17. doi: 10.1158/2767-9764.CRC-23-0277 (PMC10865976; doi:10.1158/2767-9764.CRC-23-0277)
Supplement: Supplementary Data — This file contains supplementary data for this study. [file crc-23-0277-s01.docx]

**Supplementary Data:** Detailed description of patient clinical characteristics and assays

**Detailed Clinical Characteristics**

**Patient_02**

A 29-year-old premenopausal woman was diagnosed with carcinoma of the right breast on May 9, 2015 (HPR: IDC, squamous differentiation grade III, TNBC). The patient received four cycles of NACT (cyclophosphamide + Adriamycin + flurouracil), followed by four cycles of paclitaxel + carboplatin. The patient underwent right radical mastectomy on Nov 10, 2015 (Nodes: 0/65) but had disease progression and was recruited in this study on Dec 23, 2015. The patient underwent CT-guided biopsy of the upper lobe of the right lung on Dec 21, 2015, and capecitabine tablet treatment was initiated. After three treatment cycles, the patient showed disease progression. Biopsy sample for the second progression (right chest wall nodule) was collected on Mar 1, 2016. The patient was advised to receive three cycles of gemcitabine + carboplatin, followed by response assessment. After three cycles of gemcitabine + carboplatin, the patient exhibited partial response to therapy, and three additional cycles of gemcitabine + carboplatin were advised. However, after the fourth cycle of gemcitabine + carboplatin, the patient showed disease progression. A biopsy sample for the third progression (right chest wall lesion) was collected on Mar 1, 2016. The patient was put on cyclophosphamide tablet + methotrexate tablet metronomic chemotherapy and advised to receive palliative whole brain radiotherapy (12 Gy/2 Fraction). However, the patient did not start the advised radiotherapy and expired. FFPE blocks of primary biopsy and surgical specimens were retrospectively collected from the hospital, and three snap-frozen tissue specimens representing disease progression were stored in RNAlater and kept at -80°C for future research purposes.

**Patient_04**

A 34-year-old premenopausal woman was diagnosed with carcinoma of the left breast on August 28, 2014 (HPR: IDC grade III, TNBC, Nodes: 6/25). The patient received four cycles of NACT (cyclophosphamide + adriamycin+ flurouracil), and underwent left modified mastectomy on Jan 13, 2015. Then, the patient received four cycles of adjuvant chemotherapy with paclitaxel, followed by external beam radiation therapy to the left chest wall. Subsequently, the patient had a disease-free interval of six months. However, disease progression occurred in Jan 2015, and the patient was recruited in this study on Jan 25, 2016. The patient underwent right chest wall biopsy for the first progression on Jan 27, 2016 and was advised to receive six cycles of gemicitabine + carboplatin. The patient exhibited partial response after the third cycle. However, on the first day after the fourth cycle, disease progression was found. Biopsy (chest wall) for the second progression was performed on Mar 31, 2016. The patient was put on metronomic chemotherapy (oxaliplatin + capecitabine + cyclophosphamide), but the disease progressed further, and the patient’s health deteriorated after the first cycle, and she expired. Primary biopsy and surgical specimen FFPE blocks were collected from the hospital, and two snap-frozen tissue samples were stored in RNAlater and kept at -80°C.

**Patient_07**

A 28-year-old premenopausal woman was diagnosed with carcinoma of the left breast in August 2014 (HPR: IDC III, Micropapillary Pattern TNBC). The patient received four cycles of NACT (cyclophosphamide + epirubicin + florouracil) and underwent left breast scar revision post-lumpectomy on October 31, 2014 (Node: 3/18). The patient received 12 cycles of adjuvant chemotherapy with paclitaxel, followed by external beam radiation therapy to the left chest wall. However, nine months later, the patient progressed and was recruited in this study on March 9, 2016; she underwent biopsy of the right chest wall nodule on March 10, 2016. The patient was advised to receive six cycles of gemcitabine + carboplatin + bicalutamide. After three cycles, the patient exhibited partial response to therapy and was advised to receive three more cycles of gemcitabine + carboplatin. After six cycles, the patient had disease progression. The biopsy sample for the second progression (sternal mass) was collected on August 2, 2016. The patient was advised to receive two cycles of capecitabine tablets. After three cycles of capecitabine, the patient showed disease progression. Biopsy for the third progression was performed on September 29, 2016, and the patient was advised to receive six cycles of CMF (cyclophosphamide + methotrexate + fluorouracil) chemotherapy. The patient expired the next month.

**Germline mutations in patients**

Patient_02 harbored germline (BRCA1 c.5035delC, heterozygous deletion, dbSNP rs80357896, ClinVar VCV000055358.5), which is classified as known pathogenic. This heterozygous deletion c.5035delC is in exon 17 and is predicted to cause a frameshift and immediate premature termination of the protein (p.Leu1679Ter) (1) with a length of 1678 amino acids compared with the original length of 1863 amino acids. The resultant protein is likely to lack the BRCT2 domain of the protein (2), which will likely result in loss of function. Moreover, due to the introduction of a premature stop codon, this aberrant transcript will likely be targeted by the nonsense-mediated mRNA decay (NMD) mechanism (3). The identified variant (represented as c.5154delC in the articles) has been reported in patients of Indian, Turkish and Pakistani origins affected with breast and/or ovarian cancer, and it has been classified as 'deleterious' (4-6). Patient_07 harboured germline (BRCA1 c.4676-1 G>C, heterozygous splice site, dbSNP rs80358008, ClinVar VCV000441389.5), which is predicted to be likely pathogenic by functional effect prediction tools (ASSP (7), MaxEntScan (8) and NNSPLICE (9)). BRCA1 c.4676-1G>C lies in the essential splice acceptor site in intron 14 of the BRCA1 gene and is predicted to impede splicing at the junction of intron 14 and exon 15 of the BRCA1 gene, resulting in loss of function. Two other splice site variants, c.4676-1G>T (VCV000267560.1) and c.4676-1G>A (VCV000125725.6), altering the same essential splice acceptor site as the identified variant, have been reported as 'pathogenic' in the ClinVar database for HBOC (10). These mutations in patients Patient_02 and Patient_07 were confirmed by Sanger sequencing (Supplementary figure S1).

**Copy Number Analysis**

In Patient_02, of the 342 reduced segments (2.8 GB), 210 (59%, 1.68 GB) were conserved across all three samples at progression while 132 (40%, 1.1 GB) were discordant, suggesting they were either lost or gained during evolution. Of the 206 concordant RS, 101 (51%, 1.61 GB) were copy number gains, 100 (1.02%, 31MB) were heterozygous diploid, 5 (0.99%, 30MB) were LOH, 2 (0.19%, 5KB) were homozygous deletions, and 2 (0.19%, 5KB) were CN-LOH.

In Patient_04, of the 290 (2.8 GB) reduced segments, 226 (77.45%, 2.1 GB) were concordant in the two samples and 64 (22.54%, 0.6 GB) were discordant. Of the 226 concordant reduced segments, 59 (37.77%, 1.06 GB) were copy number gains, 107 (1.0%, 28MB) were heterozygous diploid, 35 (30.09%, 0.85 GB) were LOH, 4 (8.37%, 0.2 GB) were homozygous deletions, and 21 (0.20%, 5KB) were CN-LOH.

In Patient_07, of the 372 RS (2.7 GB), 275 (59.78%, 1.6 GB) were concordant across all three samples while 97 (40.21%, 1.1 GB) were discordant. Of the 275 concordant RS, 81 (38.11%, 1.06 GB) were copy number gains, 143 (2.28%, 63MB) were heterozygous diploid, 25 (10.50%, 0.29 GB) were LOH, 6 (8.15%, 0.22GB) were homozygous deletions, and 20 (0.73%, 0.2 MB) were CN-LOH.

In Patient_02, we identified copy number gains in tumor samples obtained from all episodes of disease progression, some of which involve known cancer oncogenes, including 8q24.21 (*MYC*), 8q24.11 (*RAD21*), 12p13.31 (*CDH4*), 12p13.32 (*CCND2*) etc (Supplementary figure S5.A). Deletion of known tumor-suppressor genes at 9p21.3 (*CDKN2A*), 3p25.3 (*VHL*), 4q35.1 (*CASP3*), 17p13.1 (*TP53*) (Supplementary figure S5.B). Patient_04 showed amplifications at 8q24.21 (*MYC*), 8q24.11 (*RAD21*), 19q12 (*CCNE1*), 3q26.32 (*PIK3CA*) and deletions at 10q23.31 (*PTEN*), 4q31.3 (*CDH274*) (Supplementary figure S5.C and D). In Patient_07, we identified copy number gains at 8q24.21 (*MYC*), 12p13.31 (*CDH4*), 7q34 (*BRAF*) and deletions at 10q23.31 (*PTEN*), 9p21.3 (*CDKN2A*), 12q12 (*ARID2*) (Supplementary figure S5.E and F). The comprehensive gene list for each corresponding copy number gain and loss is provided in Supplementary Table S4.

**TNBC tumors show branching pattern of clonal evolution with a persistent stem clone**

**Patient_02**

The treatment naïve primary tumor biopsy obtained at diagnosis (02_Bio) showed 3 clones: stem clone A (Cellular Prevalence=0.01), and subclones B (CP=0.02) and D (CP=0.21). Stem clone A which persisted through the disease course contained two Tier 1 mutations (*PDGFRB* p.V761I, *COL3A1* p.S1425I), four Tier 2 mutations (*ARID2* p.S564X) and eleven Tier 3 mutations (*NECTIN3* p.T226M, *MYH13* p.N1922S). Subclone B gained one Tier 1 mutation (*AMER1* p.E191Q), two Tier 2 mutations (*BAZ1A* p.E940G, *PPFIBP1* p.A732V) and 13 Tier 3 mutations (*SI* p.M1523I, *PCDHA6* p.G97R, *HPS4* p.D603N, *CTTNBP2* p.S471P). This patient underwent neoadjuvant chemotherapy with four cycles of cyclophosphamide, doxorubicin and fluorouracil followed by four cycles of paclitaxel and carboplatin to downstage the disease. Subclone D, which was previously dominant, was completely eradicated, while stem clone A became the dominant clone at a cellular prevalence of 0.58, having undergone clonal expansion during neoadjuvant chemotherapy. Subclone B had marginally expanded (CP=0.03). This suggests that subclone D was sensitive to neoadjuvant chemotherapy while stem clone A and subclone B were resistant. The post neoadjuvant [surgical resection (02_Sur)] specimen had acquired one new subclone (Clone C) at a cellular prevalence of 0.22, which disappeared in the next progression [lung metastasis (02_Rec1)]. This patient was started on paclitaxel plus carboplatin after surgery but relapsed in the lung, lymph nodes and parasternal region after the second cycle, 50 days after surgery. Subclone C had two Tier 1 (*PRDM2* p.D76E, *KDM6A* p.R165X), two Tier 2 (*PTPRK* p.S1438T, *PREX2* p.H111Q) and seven Tier 3 mutations. The metastatic lung nodule analysis identified Clone A (CP=0.36) and subclone B (CP=0.49) as the only clones in this sample. The patient was started on oral capecitabine but experienced disease progression in the right chest wall 69 days after starting this treatment, which was biopsied (02_Rec2). There was decreased cellular prevalence of stem clone A in 02_Rec2 (0.08) compared with the surgical specimen (0.58) and lung metastasis (0.36). 02_Rec2 also showed the presence of a new subclone E with a cellular prevalence of 0.09 and its daughter subclone G with a cellular prevalence of 0.74. Functional annotation of subclone E revealed one each of Tier 1 (*BRIP1* p.E1203Q) and Tier 2 (*ALDH2* p.K404N) mutations. Subclone G was absent in the sample obtained at next progression. The patient was subsequently started on gemcitabine and cisplatin chemotherapy. She did not respond to this treatment, and another tumour sample was obtained from the right chest wall lesion after three cycles of this treatment (~100 days). This sample showed the presence of stem clone A (CP=0.04), subclone E with a cellular prevalence of 0.53), a new subclone F with a cellular prevalence of 0.44) arising from subclone E, and disappearance of subclone G. Notably, there was a reduction in the cellular prevalence of stem clone A compared with all previous samples and this clone constituted only a small fraction at this timepoint. Subclone E, first seen in the previous sample (02_Rec2), was now the dominant clone, along with its new subclone F. The patient further presented with brain metastasis and was started on palliative radiotherapy and metronomic chemotherapy, and no further tumor samples could be collected. The patient died shortly thereafter.

**Patient_04**

This patient underwent neoadjuvant chemotherapy with four cycles of cyclophosphamide, doxorubicin and fluorouracil, followed by surgery. Subclone C disappeared in the surgical resection specimen (04_Sur) with the appearance of a new subclone B with a cellular prevalence 0.16 (277 mutations). Stem Clone A, which contained one Tier 1 mutation (*TP53* p.I119S), two Tier 2 mutations (*BRAF* p.Q386L and *KMT2C* p.G4125C), and two Tier 3 mutations (*GOLGB1* p.P2841L *KDM4E* p.R240W), underwent clonal expansion with cellular prevalence 0.06. In contrast, the cellular prevalence of subclone E was reduced to 0.29.

This patient experienced disease relapse six months after surgery, which showed disappearance of subclone B and appearance of two new subclones - subclone F with a cellular prevalence of 0.59 (60 mutations, two Tier 2 and five Tier 3 mutations) and subclone G with a cellular prevalence of 0.18 (27 mutations, one Tier 1 mutation *LRP1B* p.K1694T). There was a clonal expansion of stem clone with a cellular prevalence 0.14, while there was further reduction in subclone E with a cellular prevalence of 0.07. Interestingly, subclone B, which harboured 277 mutations, of which 17 were Tier 1 mutations, disappeared and was not seen again through the remaining disease course of this patient. The patient was next treated with gemcitabine and carboplatin to which the tumour did not respond with disease progression after three cycles (04_Rec2). 04_Rec2 sample showed expansion of subclone E with cellular prevalence of 0.17 and subclone F with cellular prevalence of 0.46, reduction in stem clone A with cellular prevalence 0.11, and complete disappearance of subclone G. The patient was then treated with oral cyclophosphamide, capecitabine and oxaliplatin. Her health deteriorated after the first cycle of this treatment, and she died shortly thereafter.

**Patient_07**

This patient underwent four cycles of neoadjuvant chemotherapy with cyclophosphamide, epirubicin and fluorouracil, followed by surgery. The surgical excision sample was depleted of tissue and not available for this analysis. The patient received adjuvant post-operative chemotherapy (paclitaxel) followed by radiotherapy. She experienced a relapse in the contralateral chest wall 13 months after the last dose of chemotherapy (07_Rec1). We identified two new subclones in this sample - subclone C [gain of one Tier 2 mutation *(CDK12 p.P567R*) and six Tier 3 mutations] with a cellular prevalence of 0.49, and subclone D (gain of three Tier 2 Mutations and three Tier 3 mutations) with a cellular prevalence of 0.01, while subclone E disappeared. The patient then received gemcitabine and carboplatin but experienced disease progression in internal mammary lymph node and sternum (07_Rec2) after 6 cycles of this treatment. 07_Rec2 sample showed a more complex tumour comprised of five clones. There was a reduction in subclone B (cellular prevalence of 0.08) and subclone C (cellular prevalence of 0.14), while subclone D increased with a cellular prevalence of 0.18. The stem clone A persisted at a cellular prevalence of 0.07. A new subclone F with a cellular prevalence of 0.42 was detected in this sample, which did not show a gain of any additional Tier 1, 2 or 3 mutations but showed an additional seven Tier 4 mutations. The patient then received capecitabine, to which there was no response and further disease progression after three cycles, with scalp nodules and fungating sternal mass (07_Rec3). Subclone F persisted with a cellular prevalence of 0.24 and gave rise to a new daughter, subclone G (gain of two Tier 3 mutations), with a cellular prevalence of 0.22. Stem clone A and subclone B persisted with a cellular prevalence of 0.09 and 0.21, respectively, while subclone C had reduced substantially with cellular prevalence of 0.045. The patient was then started on oral cyclophosphamide, methotrexate and fluorouracil but died within two months.

**Circulating tumor DNA (ctDNA) captures clonal and subclonal mutations with higher sensitivity compared with tissue biopsy**

**Patient_02**

In Patient_02, the first progression was clinically detected in the lungs. Tumor tissue analysis showed subclone B (74 mutations) to be present in treatment-naïve diagnostic biopsy, surgical specimen and first recurrence sample but absent in tissue samples from two subsequent disease progressions. However, ctDNA in the subsequent two episodes of disease progression showed the presence of a majority of subclone B mutations (58 in P2R2 and 63 in P2R3). Similarly, in the tissue analysis subclone D was only identified in treatment naïve biopsy but ctDNA detected this subclone at other timepoints (P2R1=6 mutations, P2R2=1 mutation, P2R3=11 mutations), subclone C was identified only in post-neoadjuvant surgical specimen but ctDNA detected it at other timepoints (P2R1=4 mutations, P2R3=10 mutations), subclone G was detected only in Rec_2 tissue sample but ctDNA detected it at other timepoints (P2R1=2 mutations, P2R3=32 mutations), and subclone F was detected only in Rec_3 tissue sample but ctDNA detected it at other timepoints (P2R1=16 mutations, P2R2=32 mutations). Strikingly, subclone E was absent in Rec_1 lung tissue, but it's mutations were seen in the corresponding ctDNA at the time of lung relapse, indicating that the next progression was already seeded at this point of time, although it was clinically undetectable.

**Patient_04**

In Patient_04, subclone B was detected only in the surgical resection sample and absent from samples obtained at all subsequent progressions, but mutations in this subclone were identified at low VAF (<2%) in ctDNA samples at those timepoints. This suggests that tissue analysis missed subclone B due to tumour heterogeneity.

**Patient_07**

In Patient_07, the clonal structure of tumour tissue was recapitulated in ctDNA with high concordance in terms of variant allele frequency for mutations comprising stem alone A and subclones B and C. Of note, subclones D and G were detected in tumor tissue but the corresponding mutations were absent in ctDNA, while mutations from subclone F were prevalent at low allelic fractions.

**Many stem clone mutations continue to be expressed during tumor evolution**

**Patient_02**

Of the 55 stem mutations in clone A in Patient_02, 21, 26 and 25 mutations were also expressed at mRNA level in 02_Rec1, 02_Rec2 and 02_Rec3 samples, respectively. Subclones B, G and F, present exclusively in 02_Rec1, 02_Rec2 and 02_Rec3 samples, respectively, showed the expression of 21/74 mutations, 17/35 mutations, and 15/62 mutations at the mRNA level in the corresponding samples. Of the 20 mutations in subclone E, 11 and 13 mutations were expressed in 02_Rec2 and 02_Rec3, respectively.

**Patient_04**

In Patient_04, 9 of 18 stem clone A mutations were expressed in 04_Rec1 and 04_Rec2 samples, subclone B mutations (n=277) were not expressed in 04_Rec1 and 04_Rec2 samples, 6 of 27 mutations in subclone G were expressed in 04_Rec1 sample, but were not expressed in 04_Rec2, 7 of 30 mutations in subclone D were expressed in 04_Rec2 sample, and 32 of 60 mutations in subclone F were expressed in 04_Rec1 and 04_Rec2 samples.

**Patient_07**

In Patient_07, of the 41 mutations in stem clone A, 10, 18, and 10 mutations were expressed at mRNA level in 07_Rec1, 07_Rec2 and 07_Rec3 samples, respectively. Of the 42 mutations in subclone C, 18, 20, and 21 were expressed in 07_Rec1, 07_Rec2 and 07_Rec3 samples, respectively, 6 of 12 mutations in subclone D were expressed in 07_Rec2 but were absent in 07_Rec1 and 07_Rec3 samples, and 4 of 21 mutations were expressed in the late emerging subclone G in 07_Rec3 sample. Interestingly, mutations from stem clone A were more likely to be expressed at higher levels than mutations from branch subclones.

**References**

1. Findlay GM, Daza RM, Martin B, Zhang MD, Leith AP, Gasperini M, et al. Accurate classification of BRCA1 variants with saturation genome editing. Nature. 2018;562(7726):217-22.

2. Quiles F, Fernandez-Rodriguez J, Mosca R, Feliubadalo L, Tornero E, Brunet J, et al. Functional and structural analysis of C-terminal BRCA1 missense variants. PLoS One. 2013;8(4):e61302.

3. Maquat LE. Nonsense-mediated mRNA decay: splicing, translation and mRNP dynamics. Nature reviews Molecular cell biology. 2004;5(2):89-99.

4. Juwle A, Saranath D. BRCA1/BRCA2 gene mutations/SNPs and BRCA1 haplotypes in early-onset breast cancer patients of Indian ethnicity. Medical oncology. 2012;29(5):3272-81.

5. Manguoglu E, Guran S, Yamac D, Colak T, Simsek M, Baykara M, et al. Germline mutations of BRCA1 and BRCA2 genes in Turkish breast, ovarian, and prostate cancer patients. Cancer genetics and cytogenetics. 2010;203(2):230-7.

6. Rashid MU, Muhammad N, Bajwa S, Faisal S, Tahseen M, Bermejo JL, et al. High prevalence and predominance of BRCA1 germline mutations in Pakistani triple-negative breast cancer patients. BMC cancer. 2016;16(1):673.

7. Wang M, Marin A. Characterization and prediction of alternative splice sites. Gene. 2006;366(2):219-27.

8. Yeo G, Burge CB. Maximum entropy modeling of short sequence motifs with applications to RNA splicing signals. Journal of computational biology : a journal of computational molecular cell biology. 2004;11(2-3):377-94.

9. Reese MG, Eeckman FH, Kulp D, Haussler D. Improved splice site detection in Genie. Journal of computational biology : a journal of computational molecular cell biology. 1997;4(3):311-23.

10. Shao D, Cheng S, Guo F, Zhu C, Yuan Y, Hu K, et al. Prevalence of hereditary breast and ovarian cancer (HBOC) predisposition gene mutations among 882 HBOC high-risk Chinese individuals. Cancer science. 2020;111(2):647-57.
