# Supplementary material for: Natural History of Germline BRCA1 Mutated and BRCA Wild-type Triple-negative Breast Cancer
Source: Cancer Res Commun. 2024 Feb 14;4(2):404–17. doi: 10.1158/2767-9764.CRC-23-0277 (PMC10865976; doi:10.1158/2767-9764.CRC-23-0277)
Supplement: Supplementary Materials and Methods — This file contains the supplementary materials and methods for this study. [file crc-23-0277-s02.docx]

**Supplementary Materials and Methods:** Technical details of the NGS assays and bioinformatics analysis

1. DNA Based OMICS assays:
2. Whole exome capture design
3. Custom amplicon targeted NGS assay for detecting germline variants
4. Sanger sequencing validation of germline variants
5. Deep sequencing and validation using a targeted custom amplicon NGS assay
6. Whole transcriptome NGS library preparation
7. Bioinformatic analysis
8. CNV analysis using SNP arrays
9. Copy number inference from WES
10. Somatic mutation calling from i) WES and ii) RNA-Seq data
11. Germline variation calling from WBC using a targeted NGS assay
12. **DNA Based OMICS assays:**
13. **Whole exome capture design:** We used the Agilent V4+UTR (71M) capture kit for library preparation, with a read length of 150 bp, as per manufacturer’s protocol. Briefly, the qualified genomic DNA sample was randomly fragmented into fragments with a base pair peak of 150 to 200 bp; adapters were then ligated to both ends of the resulting fragments. The adapter-ligated templates were purified using the Agencourt AMPure SPRI beads, and fragments with an insert size of approximately 200 bp were extracted. The extracted DNA was amplified using ligation-mediated polymerase chain reaction (LM-PCR), purified, and hybridized to the SureSelect Biotinylated RNA Library (BAITS) for enrichment. Hybridized fragments were bound to the streptavidin beads, whereas non-hybridized fragments were washed off after 24 hr. The captured LM-PCR products were subjected to analysis using the Agilent 2100 Bioanalyzer to estimate the magnitude of enrichment.
14. **Custom amplicon targeted NGS assay for detecting germline variants.** This assay sequenced all coding exons (>97% coverage) and important splice sites for 25 genes associated with inherited cancers as recommended by the American college of medical genetics (ACMG) (Supplementary Table S8, GLCT_001 ACMG recommended), and also 19 genes known to be commonly mutated in inherited breast and ovarian cancer (Supplementary Table S8, GLCT_002 Breast and ovarian cancer). This assay has been reported to show sensitivity of 98.2%, specificity of 100% and reproducibility of 99.5% (Data not shown). 50 ng Genomic DNA (quantified using Qubit Flourometer) isolated from WBC was used for tagmentation based library preparation using Nextera Library preparation protocol as recommended by Illumina. Adaptors and Illumina Miseq Specific barcodes were incorporated using a limited cycle PCR step. The tagged and amplified sample libraries were quantified on Qubit and subjected to Q.C using Tapestation. Individual libraries were combined into a single tube and set up for enrichment. Two simultaneous enrichment steps were performed to optimize the pull down of the regions of interest using biotinylated target specific probes. Target libraries were amplified using limited PCR steps and loaded for sequencing on the illumina MiSeq to obtain a minimum of 0.3 GB data per sample.
15. **Sanger sequencing validation of germline variants:** Germline variants in BRCA1 gene were confirmed by Sanger sequencing. Briefly, Primers designed at least 60 bases upstream and downstream of the exonic region of BRCA1 exons (exon 16 and 17) and used for PCR amplification of genomic DNA extracted from peripheral blood mononuclear cells (lymphocytes) of the patients. The amplified product was subjected to Sanger sequencing after an enzymatic clean up. Sequencing was carried out on Thermo fisher Genetic analyser 3500 automated DNA sequences. Chromatograms were read with ChromasLite software after aligning with the reference sequence of BRCA1 NM 007294.3. 41
16. **Deep sequencing and validation using a targeted custom amplicon NGS assay:** Deep sequencing was performed on selected somatic mutations that were identified using exome sequencing data. For library preparation, high-quality DNA extracted from FFPE, fresh-frozen, buffy coat, and peripheral blood samples was used. An AmpliSeq custom DNA panel (<http://sapac.support.illumina.com/downloads/ampliseq-for-illumina-custom-and-community-panels-reference-guide-1000000036408.html>) was designed for library preparation. Targets were amplified using PCR (16 cycles at 99°C for 15 sec and 60°C for 4 min). The amplicons (125 bp/mutation) generated from this step were subjected to partial digestion (using the FuPa reagent), followed by indexing and cleaning using AMPureXP beads. This was followed by one more round of PCR amplification (7 cycles at 98°C for 15 sec and 64°C for 1 min) and subsequent cleaning using AMPure XP beads. Library quality was checked and deep sequencing was carried out using HiSeq2000 at ~30,000x.
17. **Whole transcriptome NGS library preparation:** Briefly, the random fragmentation of RNA was followed by conversion to double stranded cDNA. Overhang ends resulting from fragmentation into blunt ends were end repaired and ligated with Illumina adapters. Libraries were amplified using PCR and fragment sizes were validated with Bioanalyzer (Agilent) using High Sensitivity Kit. KAPPA SyBr Green Assays for qPCR (ABI 7900HT) were used for library quantification. Paired-end sequencing on Illumina HiSeq 4000 platform with read length as 150 bp was performed. The quality of the raw sequence reads was determined using FastQC.
18. **Bioinformatic analysis:**
19. **CNV analysis using SNP arrays:** Array fluorescence intensity data (CEL files) were combined using the Chromosome Analysis Suite (ChAS; version: 3.3.0.139, RRID:SCR_015626) to produce OSCHP files. The MAPD (Median of the Absolute Values of all Pairwise Differences) is a QC metric generated by ChAS and works as a global measure for variation in the microarray probes that is ideally below 0.3 but ranged between 0.19-0.23 for our samples. The ndSNPQC (SNP Quality Control of Normal Diploid Markers) measures how well genotype alleles are resolved in the microarray data and ranged between 28.02 - 38.14 (ideally ndSNPQC ≥26). We used *asmultipcf* (allele-specific multi-sample piecewise constant fitting) algorithm (1) incorporated in the Allele-Specific Copy number Analysis of Tumour (ASCAT, RRID:SCR_016868) algorithm (2) with default settings to identify commonly shared and private chromosomal breakpoints across sequential cancer samples from individual patients. Regional information was derived from CNTools (RRID:SCR_000281, <https://bioconductor.org/packages/CNTools/>).
20. **Copy number inference from WES:** Exome data were used to infer copy number information using the VarScan2 copy-number program (version 2.4.3, RRID: SCR_006849) (3). Somatic copy number changes (SCNC) were identified using tumor-normal exome sequencing data. Pileup files generated using VarScan2 were directly converted to the seqz format using the Sequenza tool (RRID:SCR_016662) (4).
21. **Somatic mutation calling from:**
22. **WES data:** Raw paired-end reads (150 bp) in the FATSTQ format were aligned to the hg19 reference genome using the bwa mem (v.0.7.16a, RRID:SCR_022192) protocol (<https://sourceforge.net/projects/bio-bwa/files/>). Unusual flag information from sample files was removed using the SAMtools (v.1.6-1, RRID:SCR_002105) fixmate program (5). Picard tools (v.2.10.0, RRID:SCR_006525) (<https://broadinstitute.github.io/picard/>) were used for the sorting and duplicate removal steps. Qualimap (RRID:SCR_001209, <http://qualimap.bioinfo.cipf.es/>), a tool used to evaluate BAM file metrics, was used to carry out further Q.C. SAMtools mpileup was used to locate non-reference positions in tumor and germline samples using the post-recalibrated bam files as input. Default settings were used in the mpileup program, except for a mapping quality of >1, and base-alignment quality computation was disabled. The resulting mileup files were inputted directly into [2] the VarScan2 somatic program (3) to identify somatic variants in the tumor. The parameters used were *--min-var-freq 0.02, --min-coverage-normal 8, --min-coverage-tumor 6, --min-coverage 8,* and *--somatic-p-value 0.05*. VarScan2 processSomatic was used to extract high confidence somatic variants using a maximum variant allele frequency of 0.05 for germline samples and minimum variant allele frequency of 0.02 for tumor samples. Further, false positive CNVs were removed using fpfilter.pl from VarScan2, which takes input metrics of read counts (generated using bam-readcount) of variants identified in the previous step. Henceforth, we restricted our analysis to the identified high confidence somatic variants and functionally annotated them using the latest version of the ANNOVAR software (RRID:SCR_012821) (6). We excluded all the variants in non-coding regions (intronic, intergenic, ncRNA, UTR etc.) and restricted further analysis to variants in coding regions (exonic and splicing). Furthermore, we excluded all synonymous and unknown-significance variants from further study and restricted our analysis to nonsynonymous variants. To account for potential artifacts in FFPE samples, we filtered the C>T/G>A mutations that were present in the final mutation pool generated from FFPE samples. Specialized filters were applied to the FFPE surgical specimens of patient 04, in which exclusive mutations (non-recurrent) with an altered depth less than 8 (AD <8) were removed. We compiled a somatic mutation list for each sample and restricted further assessments to variants that occurred in at least two samples. In addition, we preferentially selected singletons in fresh-frozen samples. From FFPE singletons, we excluded variants that were sequencing artifacts associated with the paraffinization process [13, 14].
23. **RNA-Seq data:** Raw paired-end reads (150 bp) in the FATSTQ format were converted to bam file using STAR aligner tool (RRID:SCR_004463). UCSC known genes gtf file were used to create reference index files using STAR tool. VarScan2 somatic program with default parameters were used to identify somatic variants in the tumor. Blood DNA was used as germline control.
24. **Germline variation calling from WBC using a targeted NGS assay:** The reads from the FASTQ files were aligned against the whole genome build hg19 using STRAND® NGS v3.3.5 (http://www.strand-ngs.com). Five base pairs from the 3' end of the reads were trimmed, as were 3' end bases with quality below 20. Reads which had length less than 25 bp after trimming were not considered for alignment. A maximum of 5 matches of alignment score at least 90% with a gap percentage of 45 were computed. Reads that failed QC (quality control), reads with average quality less than 20, reads with ambiguous characters and reads which are duplicates were all filtered out. The reads were realigned using the local realignment tool in STRAND® NGS. The reads with alignment score less than 95% and partially aligned reads were all filtered out. The STRAND® NGS variant caller was used to detect variants at locations in the target regions covered by a minimum of 10 reads with at least 2 variant reads. Mate missing reads were ignored while calling the variants. Variants with a decibel score of at least 50 were reported. Variants with a Strand Bias ≥100% and with a Total Reads ≥50, and InDel variants in homopolymer stretches ≥12 bp long with supporting reads ≤90% were filtered out. Variants were then imported into StrandOmics. Annotation and prioritization of variants was done by automated pipelines in StrandOmics. The StrandOmics user interface was then used for identifying variants of interest and for reporting these variants. All variants reported were verified to have good raw read quality using the STRAND® NGS genome browser.
25. **Deep sequencing and validation using a targeted custom amplicon NGS assay:** Raw paired-end reads (150 bp) in the FATSTQ format were aligned to the hg19 reference genome using bwa mem (<https://sourceforge.net/projects/bio-bwa/files/>). Unusual flag information from sample files was removed using the SAMtools fixmate program. Picard tools were used for sorting. The deepSNV Bioconductor package (RRID:SCR_006214) was used to compute the total depth and altered depth of each mutation from the ultradeep sequencing data. Variants were termed significant if the p-value computed by deepSNV for the likelihood ratio test (Benjamini Hochberg corrected) <0.001 and the observed allele frequency for the altered base was greater than 10. Significant variants were visualized as two groups based on their allele frequency (Red, >2% and Blue, <2%). We categorized the variants chosen to be validated by ultra-high depth sequencing data (n=1006) into four tiers. Tier 1 variants were those with an associated ID in Catalogue Of Somatic Mutations In Cancer (COSMIC, (7-9), RRID:SCR_002260) or an ID in International Cancer Genome Consortium (ICGC, (10,11), RRID:SCR_021722) and whose genes were present in the COSMIC Cancer Census Gene downloaded from COSMIC (v92.) Database on May 1, 2021. Tier 2 variants were located in genes in the COSMIC Cancer Census Gene database, which did not have an associated COSMIC ID or an ICGC ID. Tier 3 variants were in genes with an associated COSMIC ID or an ICGC ID, which were absent in the COSMIC Cancer Census Gene Database. Tier 4 variants were novel, did not have any associated COSMIC ID or ICGC ID, and whose genes were absent in the COSMIC Cancer Census Gene Database. Presumably, these variants are entirely novel with respect to their role in cancer or population-specific alleles.

**References**

1. Ross EM, Haase K, Van Loo P, Markowetz F. Allele-specific multi-sample copy number segmentation in ASCAT. Bioinformatics **2021**;37:1909-11

2. Van Loo P, Nordgard SH, Lingjaerde OC, Russnes HG, Rye IH, Sun W*, et al.* Allele-specific copy number analysis of tumors. Proceedings of the National Academy of Sciences of the United States of America **2010**;107:16910-5

3. Koboldt DC, Zhang Q, Larson DE, Shen D, McLellan MD, Lin L*, et al.* VarScan 2: somatic mutation and copy number alteration discovery in cancer by exome sequencing. Genome research **2012**;22:568-76

4. Favero F, Joshi T, Marquard AM, Birkbak NJ, Krzystanek M, Li Q*, et al.* Sequenza: allele-specific copy number and mutation profiles from tumor sequencing data. Ann Oncol **2015**;26:64-70

5. Li H, Handsaker B, Wysoker A, Fennell T, Ruan J, Homer N*, et al.* The Sequence Alignment/Map format and SAMtools. Bioinformatics **2009**;25:2078-9

6. Wang K, Li M, Hakonarson H. ANNOVAR: functional annotation of genetic variants from high-throughput sequencing data. Nucleic acids research **2010**;38:e164

7. Bamford S, Dawson E, Forbes S, Clements J, Pettett R, Dogan A*, et al.* The COSMIC (Catalogue of Somatic Mutations in Cancer) database and website. Br J Cancer **2004**;91:355-8

8. Forbes SA, Bindal N, Bamford S, Cole C, Kok CY, Beare D*, et al.* COSMIC: mining complete cancer genomes in the Catalogue of Somatic Mutations in Cancer. Nucleic acids research **2011**;39:D945-50

9. Tate JG, Bamford S, Jubb HC, Sondka Z, Beare DM, Bindal N*, et al.* COSMIC: the Catalogue Of Somatic Mutations In Cancer. Nucleic acids research **2019**;47:D941-D7

10. Zhang J, Bajari R, Andric D, Gerthoffert F, Lepsa A, Nahal-Bose H*, et al.* The International Cancer Genome Consortium Data Portal. Nat Biotechnol **2019**;37:367-9

11. Zhang J, Baran J, Cros A, Guberman JM, Haider S, Hsu J*, et al.* International Cancer Genome Consortium Data Portal--a one-stop shop for cancer genomics data. Database (Oxford) **2011**;2011:bar026
