# Supplementary material for: Natural History of Germline BRCA1 Mutated and BRCA Wild-type Triple-negative Breast Cancer
Source: Cancer Res Commun. 2024 Feb 14;4(2):404–17. doi: 10.1158/2767-9764.CRC-23-0277 (PMC10865976; doi:10.1158/2767-9764.CRC-23-0277)

**Supplementary Figure S1.** Sanger sequencing of germline variants identified from targeted germline NGS assay.


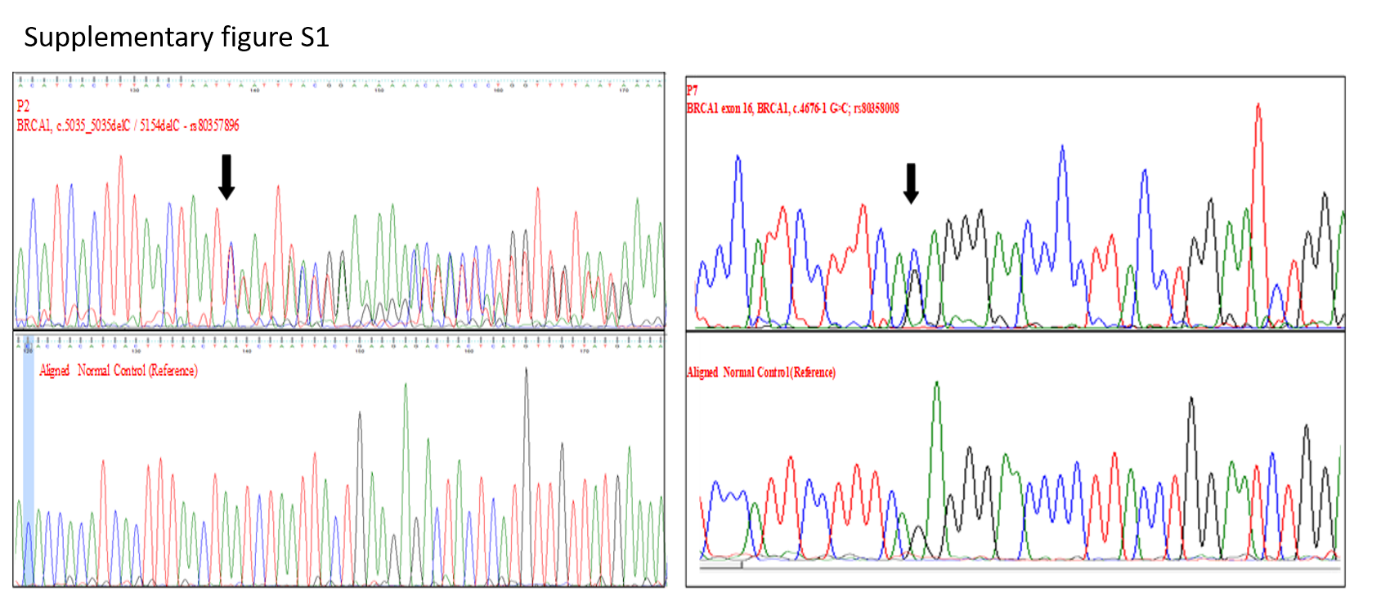

Supplement: Supplementary figure S1 — This figure shows peaks from Sanger sequencing of germline variants identified in two patient samples. [file crc-23-0277-s03.docx]
