# Supplementary material for: Natural History of Germline BRCA1 Mutated and BRCA Wild-type Triple-negative Breast Cancer
Source: Cancer Res Commun. 2024 Feb 14;4(2):404–17. doi: 10.1158/2767-9764.CRC-23-0277 (PMC10865976; doi:10.1158/2767-9764.CRC-23-0277)

**Supplementary Figure S2.** Correlation plot of variant allele frequency inferred from deep sequencing (X-axis) and exome (Y-axis). Sample names are written on the top of the chart with correlation values in parentheses. Each dot is an independent mutation.


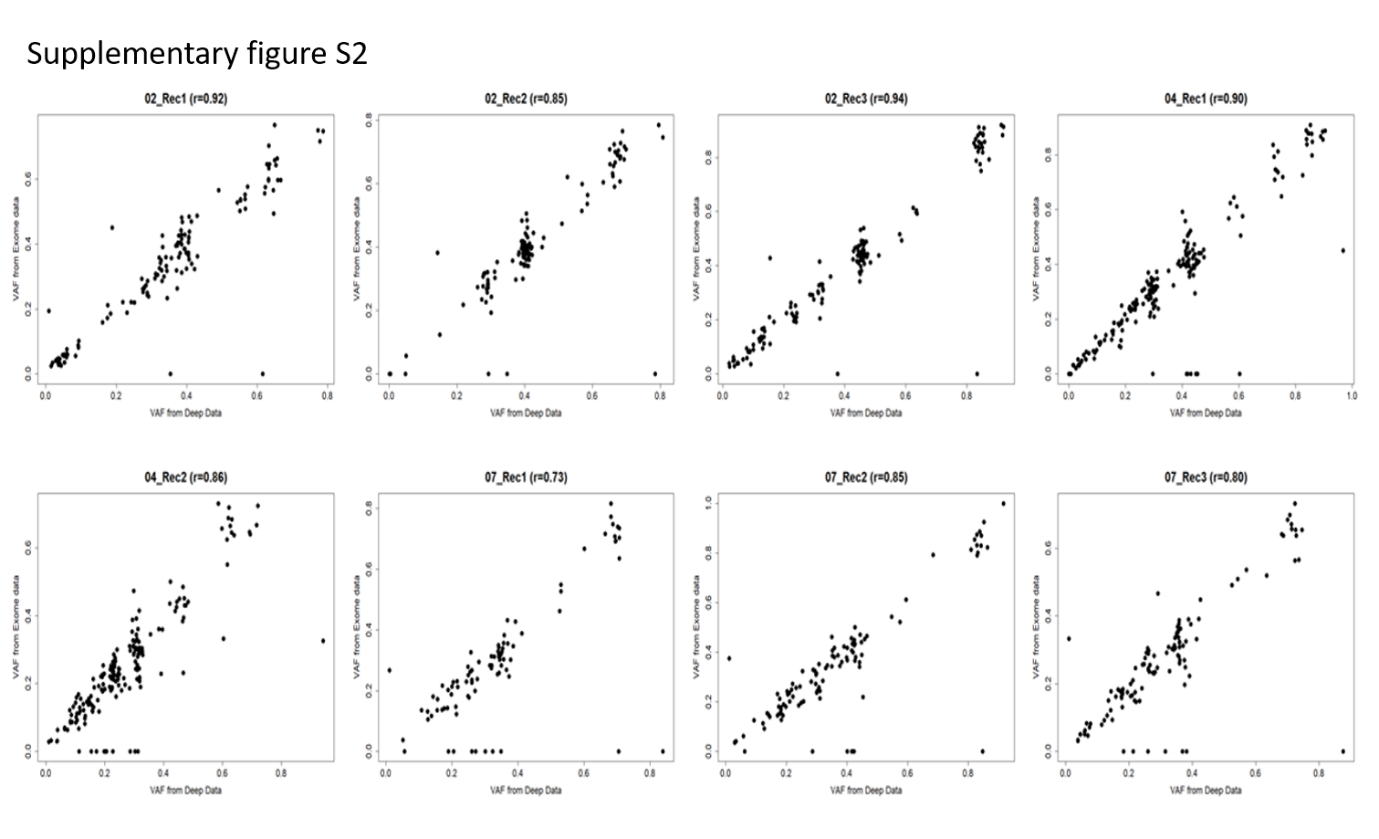

Supplement: Supplementary figure S2 — This figure describes Co-relation of VAFs for deep-sequencing of Whole Exome Sequencing data from tumour samples. [file crc-23-0277-s04.docx]
