# Supplementary material for: Natural History of Germline BRCA1 Mutated and BRCA Wild-type Triple-negative Breast Cancer
Source: Cancer Res Commun. 2024 Feb 14;4(2):404–17. doi: 10.1158/2767-9764.CRC-23-0277 (PMC10865976; doi:10.1158/2767-9764.CRC-23-0277)

**Supplementary Figure S3.** Comparison of somatic point mutations between whole exome sequencing and ultra-deep sequencing in tissues.


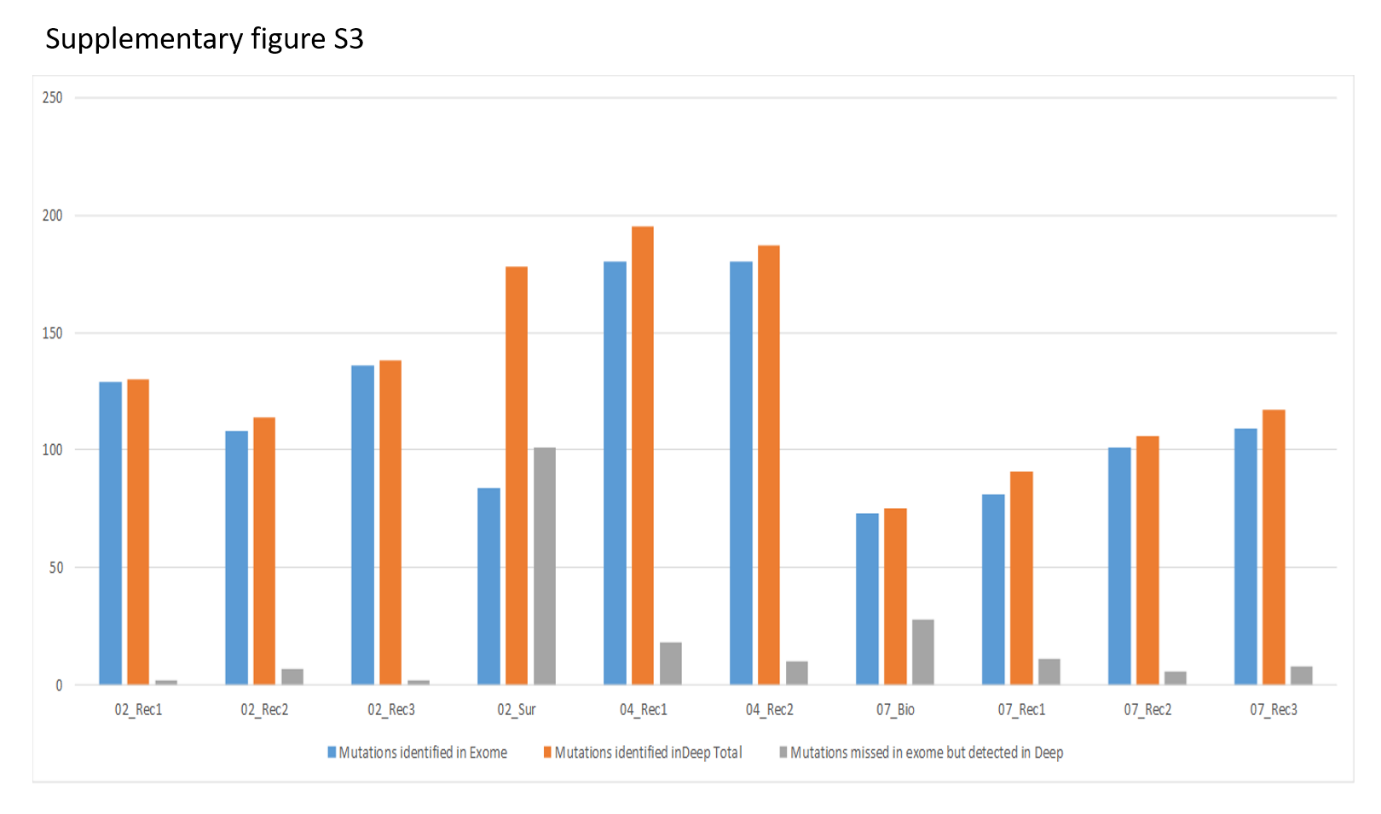

Supplement: Supplementary figure S3 — This figure shows the distribution of point mutations identified in WES and ultra-deep Sequencing. [file crc-23-0277-s05.docx]
