# Supplementary material for: Natural History of Germline BRCA1 Mutated and BRCA Wild-type Triple-negative Breast Cancer
Source: Cancer Res Commun. 2024 Feb 14;4(2):404–17. doi: 10.1158/2767-9764.CRC-23-0277 (PMC10865976; doi:10.1158/2767-9764.CRC-23-0277)

**Supplementary Figure S4.** ASCAT profile of each sample. Ploidy and tumour content (aberrant cell fraction) are written on the upper side of each figure. Allele-specific copy numbers are on Y-axis and genomic coordinates are on the X-axis. Green and red colour indicate low and high copy numbers, respectively.


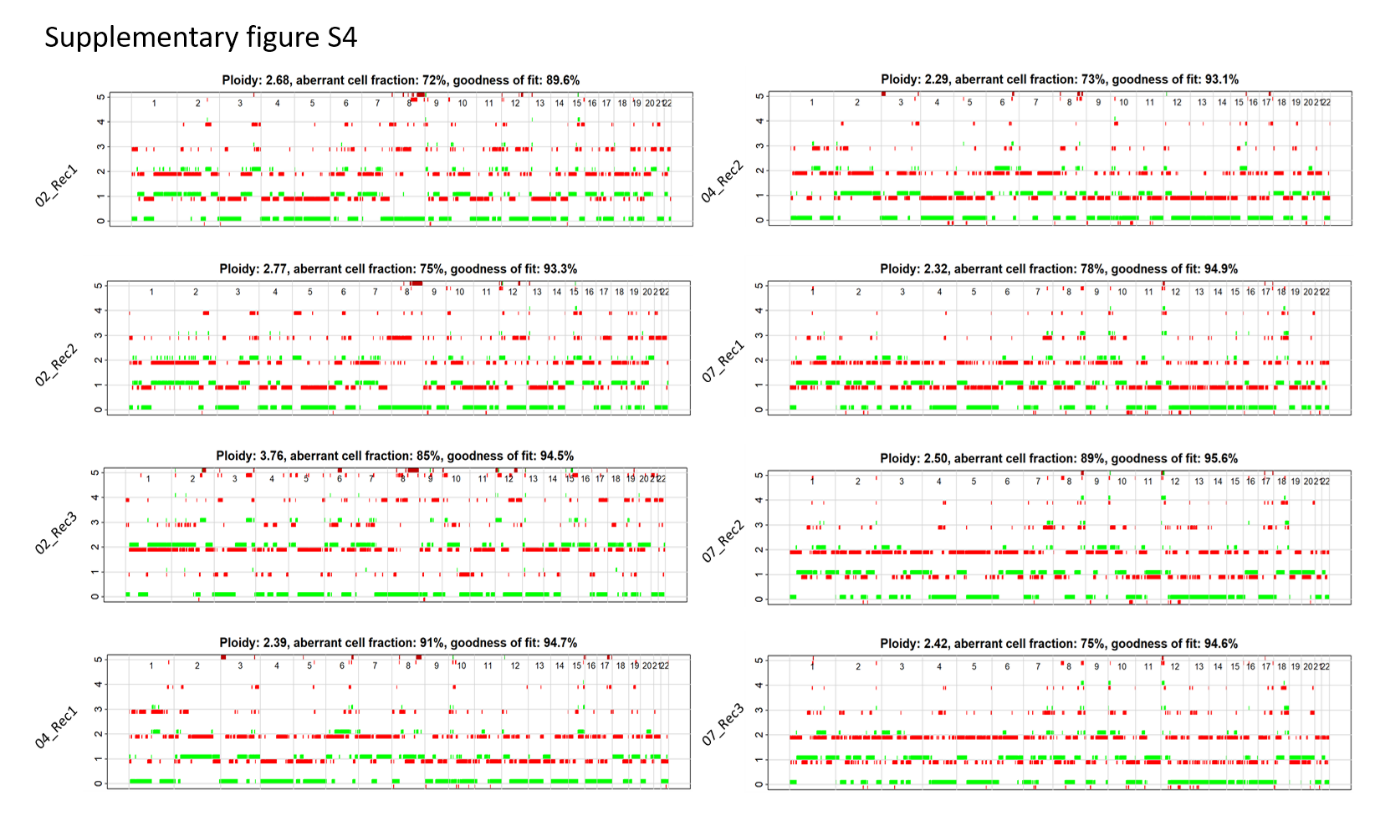

Supplement: Supplementary figure S4 — This figure shows ASCAT copy number profile of each sample. [file crc-23-0277-s06.docx]
