# Supplementary material for: Natural History of Germline BRCA1 Mutated and BRCA Wild-type Triple-negative Breast Cancer
Source: Cancer Res Commun. 2024 Feb 14;4(2):404–17. doi: 10.1158/2767-9764.CRC-23-0277 (PMC10865976; doi:10.1158/2767-9764.CRC-23-0277)

**Supplementary Figure S5.** Copy number SGOL (Segment Gain or Loss) score from all samples at disease progression in each patient is plotted with copy gains (red) and losses (blues), respectively. Key cancer-relevant genes are shown next to them. A and B. Copy gains and losses in Patient_02. C and D. Copy gains and losses in Patient_04. E and F. Copy gains and losses in Patient_07.


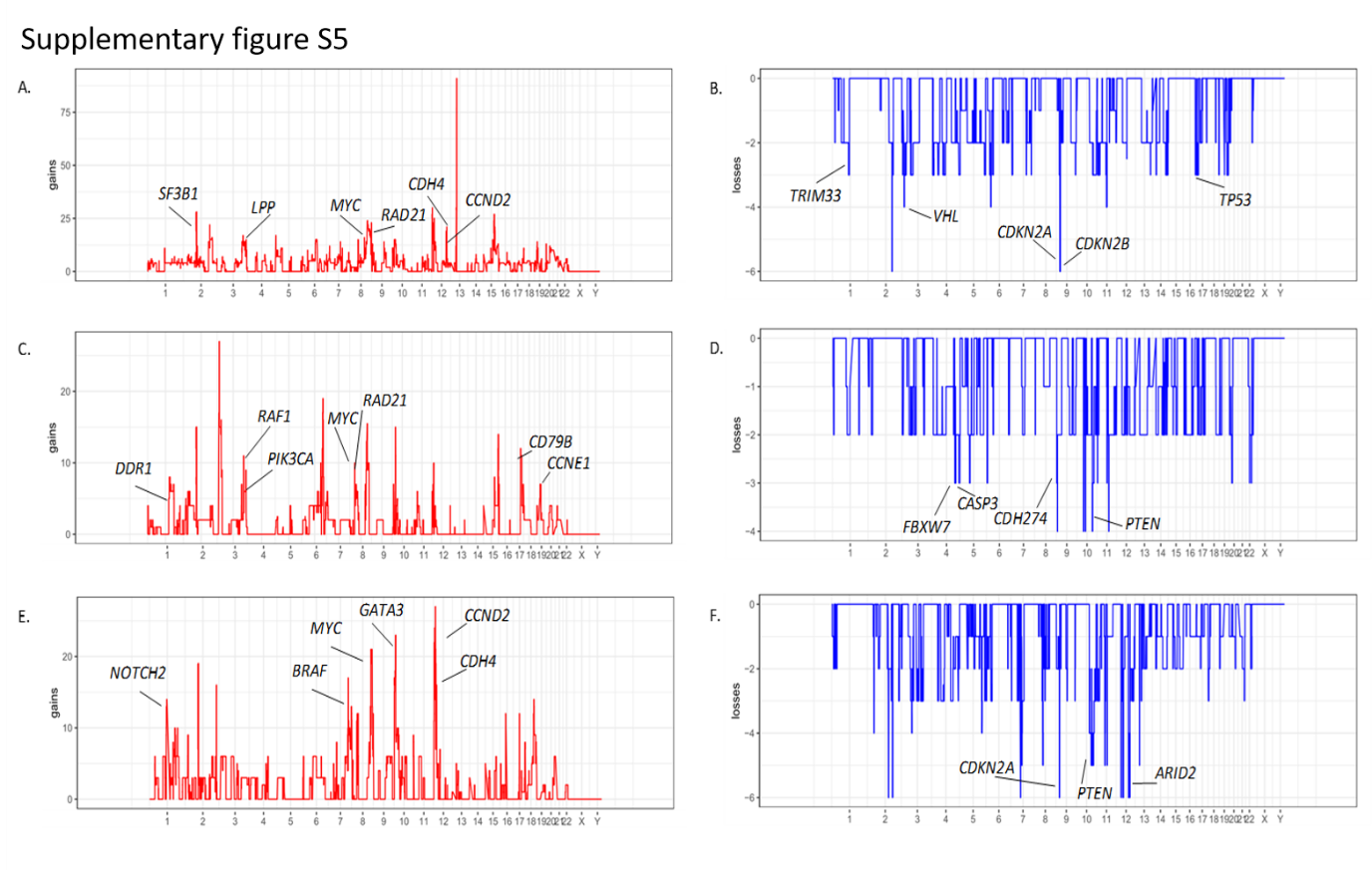

Supplement: Supplementary figure S5 — This figure shows the SGOL score for all patient samples with clinically relevant genes. [file crc-23-0277-s07.docx]
